# Forecasting the strength of preplaced aggregate concrete using interpretable machine learning approaches

**DOI:** 10.1038/s41598-024-57896-0

**Published:** 2024-04-10

**Authors:** Muhammad Faisal Javed, Muhammad Fawad, Rida Lodhi, Taoufik Najeh, Yaser Gamil

**Affiliations:** 1 Silesian University of Technology Poland, Gliwice, Poland; 2 Budapest University of Technology and Economics Hungary, Budapest, Hungary; 3 Department of Urban and Regional Planning, National University of Sciences and Technology (NUST), Islamabad, Pakistan; 4 Operation and Maintenance, Operation, Maintenance and Acoustics, Department of Civil, Environmental and Natural Resources Engineering, Lulea University of Technology, Lulea, Sweden; 5 Department of Civil Engineering, School of Engineering, Monash University Malaysia, Jalan Lagoon Selatan, 47500 Bandar Sunway, Selangor Malaysia; 6 Present Address: Department of Civil Engineering, Ghulam Ishaq Khan Institute of Engineering Sciences and Technology, Topi, Swabi, 23640 Pakistan

**Keywords:** Preplaced aggregate concrete, Two-stage concrete, Compressive strength prediction, Machine learning models, Construction engineering, Engineering, Civil engineering

## Abstract

Preplaced aggregate concrete (PAC) also known as two-stage concrete (TSC) is widely used in construction engineering for various applications. To produce PAC, a mixture of Portland cement, sand, and admixtures is injected into a mold subsequent to the deposition of coarse aggregate. This process complicates the prediction of compressive strength (CS), demanding thorough investigation. Consequently, the emphasis of this study is on enhancing the comprehension of PAC compressive strength using machine learning models. Thirteen models are evaluated with 261 data points and eleven input variables. The result depicts that xgboost demonstrates exceptional accuracy with a correlation coefficient of 0.9791 and a normalized coefficient of determination (R^2^) of 0.9583. Moreover, Gradient boosting (GB) and Cat boost (CB) also perform well due to its robust performance. In addition, Adaboost, Voting regressor, and Random forest yield precise predictions with low mean absolute error (MAE) and root mean square error (RMSE) values. The sensitivity analysis (SA) reveals the significant impact of key input parameters on overall model sensitivity. Notably, gravel takes the lead with a substantial 44.7% contribution, followed by sand at 19.5%, cement at 15.6%, and Fly ash and GGBS at 5.9% and 5.1%, respectively. The best fit model i.e., XG-Boost model, was employed for SHAP analysis to assess the relative importance of contributing attributes and optimize input variables. The SHAP analysis unveiled the water-to-binder (W/B) ratio, superplasticizer, and gravel as the most significant factors influencing the CS of PAC. Furthermore, graphical user interface (GUI) have been developed for practical applications in predicting concrete strength. This simplifies the process and offers a valuable tool for leveraging the model's potential in the field of civil engineering. This comprehensive evaluation provides valuable insights to researchers and practitioners, empowering them to make informed choices in predicting PAC compressive strength in construction projects. By enhancing the reliability and applicability of predictive models, this study contributes to the field of preplaced aggregate concrete strength prediction.

## Introduction

Concrete is a complex artificial material that consists of multiple phases, including coarse aggregate, mortar, and interfacial transition zones (ITZ). Among these components, coarse aggregate an inert material serves as the foundational skeleton that can enhance concrete's strength. It is typically a structural unit with superior strength, excellent volume stability, and remarkable durability. Thus, performing a central role in fortifying the overall integrity of the concrete structure^1^. Traditionally, the preparation of concrete involves the meticulous blending of cementing material that comprises different ingredients including cement, aggregates, and water in specific proportions. Afterward, these mixtures then go through a sequence of procedures, involving casting, curing, and molding at specific room temperature. However, in engineering applications, it is imperative to maintain the optimal workability of fresh concrete. Consequently, the amount of coarse aggregate is usually restricted to a minimal level, commonly not exceeding 1300 kg/m^3^^2^ . This limitation is particularly evident in high-performance concrete (HPC) and precast concrete, where an excess of paste exists. Hence, leading to an aggregate void age of up to 35%. In such scenarios, the void age of coarse aggregate might reach as high as 60%^2^. Due to the significant void spaces within, the coarse aggregate in this concrete variant is dispersed unevenly. Thus, lacking adequate lapping or interlocking. Consequently, the coarse aggregate fails to fulfill its intended function as a structural framework. Such concrete is susceptible to segregation and water bleeding during transportation or vibration, inevitably resulting in early cracking^3^. Additionally, the elevated proportion of cement paste in this concrete type can expedite the occurrence of early cracks. Therefore, impairing its long-term performance. Simultaneously, the inadequate utilization of a substantial quantity of cement paste hampers the economic efficiency of the concrete mixture^2,4^.

Preplaced-aggregate concrete (PAC) stands out as a distinctive concrete variant where coarse aggregates are strategically positioned within a mold, and subsequently filled with cement grout to occupy the voids between the aggregates. This specialized type of concrete finds noteworthy applications in heavyweight contexts, such as high-density concrete^5^, cavity conditions^6^, marine structures^7^, and the restoration of concrete structures with minimal volume alteration^8^. Typically, the introduction of mortar into the mold can be executed either through gravitational means or by employing an injection process^9^. PAC diverges significantly from standard concrete in various key aspects. Unlike conventional concrete, where all components are uniformly mixed, PAC exhibits a distinctive structure. In PAC, mortar particles are enclosed within the interstices of coarse aggregate materials. Thus, enabling gravel grains to establish direct physical contact with one another. This unique arrangement contrasts with the homogenous mixture found in traditional concrete formulations^10^. Secondly, a notable characteristic of PAC lies in its composition with approximately 60% of the PAC volume occupied by coarse aggregate particles. While the remaining 40% is filled with grout. This composition underscores the substantial presence of coarse aggregates, indicating a distinctive feature of PAC in contrast to conventional concrete formulations^11^. Due to these factors, the transmission of stresses in PAC occurs predominantly through the contact regions between its coarse aggregate particles^12^. Moreover, it is also possible to utilize recycled aggregates or steel slag aggregates instead of coarse aggregate particles^13^. Thus, fly ash can serve as a viable binder material when incorporated into PAC. Substituting fly ash for cement has been observed to enhance the workability and fluidity of PAC grout^14,15^. The incorporation of fly ash, an industrial byproduct, into PAC offers the potential to reduce cement usage by up to 10%^16^. Furthermore, the inclusion of fly ash provides an opportunity to decrease the density of PAC without compromising its compressive and bending strength^17^. In addition, scholars conducted a comprehensive investigation into the incorporation of silica fume (SF) in PAC to check its utmost effect in concrete^18^. The findings demonstrated a notable enhancement in the strength characteristics of PAC attributed to the utilization of silica fume^18,19^. Furthermore, superplasticizers stand out as the predominant additives employed in PAC grout. This preference arises from the fundamental requirement of filling the gapes among partials of coarse aggregate with grout. Therefore, a crucial step in PAC construction. Incorporating a superplasticizer augments the fluidity of the grout, simplifying the injection process into the mold. Research outcomes indicate that the concurrent application of superplasticizers with additional additives, including silica fume, expanding admixture and fly ash significantly to enhancing the mechanical properties of PAC^9,20^.

Preplaced-Aggregate Concrete (PAC) finds diverse applications, one of which involves the construction of submerged concrete structures^7^, renovation of existing concrete structures^6^, mass concreting^5^, construction of nuclear power plants^8^, and structures involving complex reinforcement^6^. Since its inception, PAC has found extensive application in a multitude of global projects. Notably, it was utilized in the preliminary cladding of Barker Dam in Nederland, Colorado, in the year 1946, highlighting its early deployment and effectiveness in significant engineering endeavors^21^. In 1951, it was also employed within the scroll case at Bull Dam Powerhouse^8^, and in Auxiliary dam in the year 2006, located in China^22^. Due to the higher proportion of coarse aggregate and lower cement content in PAC, there is potential for a reduced demand for cement. Additionally, PAC exhibits lower heat generation during the hydration process compared to conventional concrete^5^. Consequently, PAC emerges as a potentially eco-friendly substitute for conventional concrete. Further reduction in PAC's cement content can be achieved by incorporating waste supplementary cementitious materials (SCMs). These materials play a pivotal role in enhancing the concrete's durability and mechanical properties, thereby contributing to sustainable construction practices^23^. The strategic utilization of these SCMs holds the potential to not only enhance the eco-friendliness and cost-effectiveness of concrete production but also significantly decrease the carbon footprint associated with concrete manufacturing^24^. In addition, PAC exhibited superior performance compared to ordinary concrete in essential structural properties, including CS, TS, Young’s modulus, Schmidt hammer rebound number and ultrasonic pulse velocity (UPV)^12^. Besides, the splitting tensile strength, cubic compressive strength, and elastic modulus of preplaced aggregate concrete exhibited a decrease with the increase in the water-to-binder and sand-to-binder ratios^25^. Notably, in comparison to conventional concrete at equivalent compressive strengths, PAC displayed a potential increase in its elastic modulus by as much as 20%. In a study conducted by Salaimanimagudam et al.^26^ an investigation was carried out to assess the effectiveness of a topology-optimized hammerhead pier concrete beam constructed using the PAC methodology.The incorporation of steel fibers into PAC substantially enhanced its capacity to withstand impact loads. Thus, resulting in an increase ranging from 22 to 40 times, signifying a significant improvement in its impact resistance characteristics. According to Alfayez et al.^27^ the incorporation of tire rubber in preplaced aggregate concrete adversely affected its performance. Quality control of PAC through experimental testing involves considerable costs, necessitating specialized laboratory equipment, skilled workforce, and technicians. Furthermore, the conventional use of cement, sand, and fillers in PAC leads to irreparable environmental damage, with residual PAC samples post-testing posing an additional environmental concern. To mitigate these challenges, decreasing the number of experimental tests becomes imperative, allowing engineers to incorporate PAC in structures while minimizing the financial burden on projects. In response to the expanding utilization of concrete in construction projects, a notable increase in the use of Polycarboxylate-based Superplasticizers (PAC) is anticipated in the coming years. Despite the manifold benefits PACs offer their exploration in research investigations has been relatively limited. A study revealed that the optimal mixture for PAC development involves a water-to-cement ratio of 0.47 and a cement-sand ratio of 1, yielding superior mortar quality^28^. Researchers have observed a linear connection between PAC compressive strength (CS) and various characteristics of PAC mortar, achieved through the use of admixtures, silica fume, and superplasticizers^29,30^. This implies that an enhancement in mortar strength leads to a corresponding increase in concrete strength^30^. The efficiency of the mortar diminishes with increased sand content, while PAC's mechanical strength metrics continue to rise with augmented sand quantity. Additionally, higher sand grading escalates mortar viscosity. The potential reduction in mortar effectiveness due to supplementary sand can be compensated by introducing fly ash into the mixture^14,31^. PAC dictates that the mortar sand-to-cement ratio remains below 1.5^32^. Sand grading significantly influences the geo-mechanical characteristics of concrete aggregate in PAC, primarily due to the substantial sand content in the PAC mold. PAC outperforms traditional concrete in terms of both economic viability and geo-mechanical properties, with a lower fracture risk^14,33^. PAC finds application not only in high-volume concreting but also in underwater concreting^34^, marking critical applications for this material.

Contrary to the extensive research on conventional concrete, PAC has been the subject of relatively limited study. To enhance mixture qualities, Coo and Pheeraphan^14^ employed statistical approaches, albeit constrained by the limited number of admixtures applicable. Recognizing this limitation, researchers have explored Machine Learning (ML) techniques for predicting concrete CS^35,36^ and integrating concrete strength into structural modeling^37^. ML, as the practice of instructing a machine to perform prediction tasks using appropriately prepared datasets and algorithms, has gained prominence in structural engineering. Numerous articles have addressed the intersection of concrete structures and ML techniques within the past 5 years^10,38^, reflecting the increasing integration of artificial intelligence (AI) in this domain. Several machine learning (ML) algorithms, notably utilized by previous researchers, include the artificial neural network (ANN) for predicting the mechanical characteristics of concrete^39–43^, gene expression programming (GEP)^44–46^, random forest (RF)^47,48^, and multi expression programming (MEP)^53^. These models are commonly applied to large datasets, which are segmented into smaller subsets for the purposes of training, validation, and testing. To mitigate overfitting, the training process is suspended when errors escalate. Subsequently, the model is assessed using validation data to gauge its alignment with the training set. Table 1 provides a summary comparing previous studies utilizing machine learning approaches to forecast various concrete properties, highlighting the evolution and diverse methodologies employed in the field.

**Table 1:** Previous literature review.

Sr. No.	Model	Predicted properties	Material Used	Data Points	References
1	RF and GEP	CS	RHAC	192	^47^
2	GEP	CS	GGBS	351	^44^
3	BAS-RF	CS	FAGGBSFA	131	^49^
4	DT and AR	CS	Waste glass	117	^50^
5	GEP and DT	CS	FA	270	^51^
6	ANN	CS	FA	69	^39^
7	RSM and GEP	CS	Steel fibres	108	^52^
8	RF and GEP	CS	-	357	^48^
9	ANN	CS	FA	114	^40^
10	GEP, ANN, DT	Surface Chloride Concentration	FA	642	^40^
11	GEP, PSO, ANN	Bearing capacity of concrete	-	303	^41^
12	ANN	CS	RHAC	192	^42^
13	GEP, MEP	CS	Plastic waste	135	^53^
14	SVM, BR	CS	Eggshell	117	^54^
15	GEP, MNLR, RSM	CS	Fly ash	750	^55^
16	GEP	CS	Plastic waste	135	^45^
17	GEP	CS	-	357	^46^
18	GEP	CS	NZ (Natural Zeolite)	54	^56^
19	MLR, DR, MLPNN, SVR, RF	CS	FA	1030	^57^
20	ANN	FS	RHAC	35	^43^

This study developed 13 ML techniques for predicting the compressive strength of PAC. Notably, the study aims to streamline the prediction process, ensuring accurate results without extensive experimental trials. To contextualize this research, statistical, sensitivity, SHAP analysis, and the development of a graphical user interface are conducted. These additional components contribute to a thorough understanding of the model's efficacy and enhance its applicability in practical scenarios.

## Overview of machine learning (ML) models

### Linear regression

**Figure 1:**
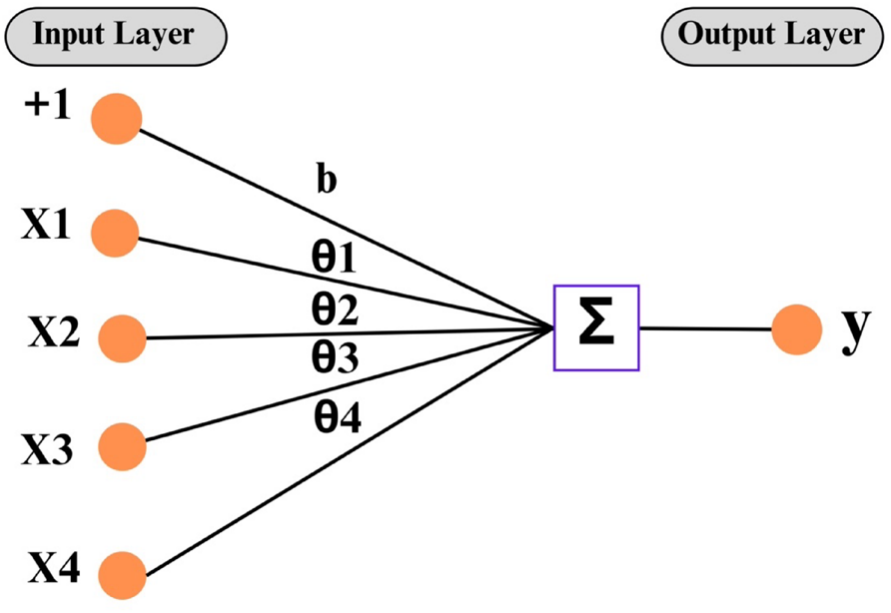
Graphical presentation of linear regression.

LR, a time-honored statistical approach, serves as a method to assess the relationship among two and more than two variables^58^. Upon establishing the correlation between input and output variables, the learning process is initiated with the objective of minimizing the loss function value, such as MSE. The variables yielding the minimum loss function value represent the ideal variables for the regression model. Despite its simplicity, LR tends to exhibit relatively modest accuracy. Equation (1) showed the general form of multiple linear regression (MLR)^59^.

1 $$\widehat{y}= {a}_{o}+ \sum_{j=1}^{m}{a}_{j}{X}_{j}$$

where $\widehat{y}$ is the predicted result, ${X}_{j}$ are the features (input) of the dataset, and *a*_0_, *a*_1_, …, *a*_*m*_ are the parameters to train. Figure 1 illustrate the graphical presentation of linear regression.

### Ridge regression

Ridge regression, introduced by Hoerl and Kennard in 1970, represents a generalization of the ordinary least squares method by incorporating a regularization term into the optimization problem. This enhancement transforms the optimization problem into

2 $${b}^{*}= {argmin}_{b} |\left|y-Xb\right|{|}_{2}^{2} + \lambda ||b|{|}_{2}^{2}$$

**Figure 2:**
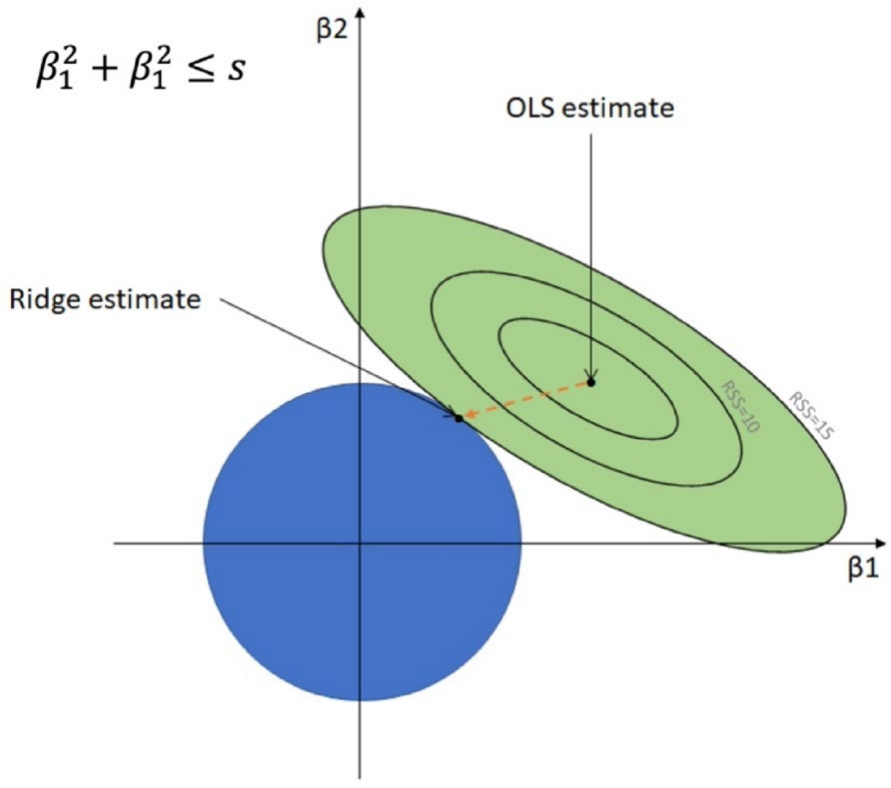
Graphical presentation of ridge regression.

Equation (2) is the objective function for Ridge Regression. The symbol ${b}^{*}$ denotes the coefficients estimated to minimize the objective function. $|\left|y-Xb\right|{|}_{2}^{2}$ represents the sum of squared errors, indicating the difference between the predicted values (Xb) and the actual values (y). $||b|{|}_{2}^{2}$ corresponds to the L2 norm (Euclidean norm) of the coefficient vector b. The parameter $\lambda$ serves as the regularization term, influencing the balance between effectively fitting the data and restraining the magnitude of the coefficients. where λ > 0, plays a pivotal role in regulating the strength of regularization. This regularization technique enhances the model's robustness by introducing a positive value λ to all eigenvalues **σ**I ≥ 0 of the matrix X⊤ X before performing matrix inversion. By employing the modified term **σ**I + λ for inversion, as opposed to **σ**I alone, the method mitigates instability stemming from small eigenvalues, making it viable for inverting underdetermined systems, where **σ**I equals zero. Instances of small eigenvalues and underdetermined systems frequently arise in scenarios involving a large number of features. In ridge regression, the regularization hyperparameter λ necessitates estimation from the data. If the estimated value of λ is too low, the model tends to overfit to noise within the data. Conversely, if the estimated value is excessively high, the model's predictive capabilities are compromised^60^. The appropriate regularization parameter is typically determined through a meticulous process involving grid search coupled with cross-validation techniques^61^. Initially, a predefined set of hyperparameter candidates (λ) is delineated, typically spanning multiple orders of magnitude and distributed over a logarithmically spaced grid. Subsequently, the dataset is partitioned into a training set (Xtrain, Ytrain) and a validation set (Xval, Yval). The regression model is trained on the training set, and its predictive performance is assessed on the validation set. This iterative process is executed for all the designated hyperparameter candidates. The candidate configuration yielding the highest prediction accuracy is chosen as the optimal selection. The ridge regression model is fitted on the entire dataset employing the selected optimal hyperparameter configuration, ensuring a comprehensive and rigorous approach to model optimization. Graphical presentation of ridge regression is depicted in Fig. 2.

### Lasso regression

The Lasso Regression technique relies on the linear predictive model depicted in Eq. (3).

3 $${\text{y}}\left({\text{x}}\right)= {\upbeta }_{{\text{o}}}+ \sum_{{\text{n}}=1}^{{\text{N}}}{\upbeta }_{{\text{o}}}{{\text{x}}}_{{\text{n}}}$$

**Figure 3:**
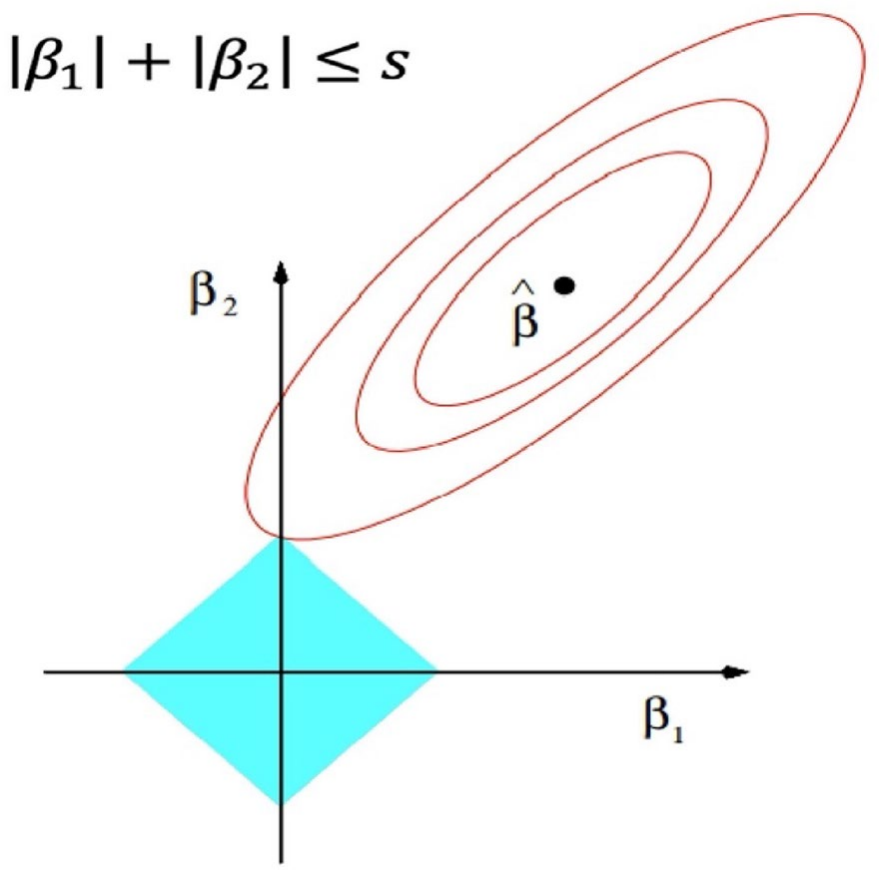
Graphical presentation of Lasso regression.

The coefficients (${\beta }_{o}$) in Eq. (3) are determined by minimizing the objective function depicted in Eq. (4). The inclusion of the L1 norm term in Eq. (4), evaluating the norm of the coefficient vector, results in certain coefficients corresponding to less significant input variables being zeroed out. This results in a predictive model that relies on a reduced set of variables, creating what is commonly referred to as a sparse model^62^. Figure 3 shows the graphical presentation of lasso regression.

4 $$\sum_{i=1}^{n}{\left[{p}_{i}- {\beta }_{o}-\sum_{j=1}^{N}{\beta }_{j}{x}_{ij} \right]}^{2}+\lambda \sum_{j=1}^{N}|{\beta }_{j}|$$

### Support vector machine

**Figure 4:**
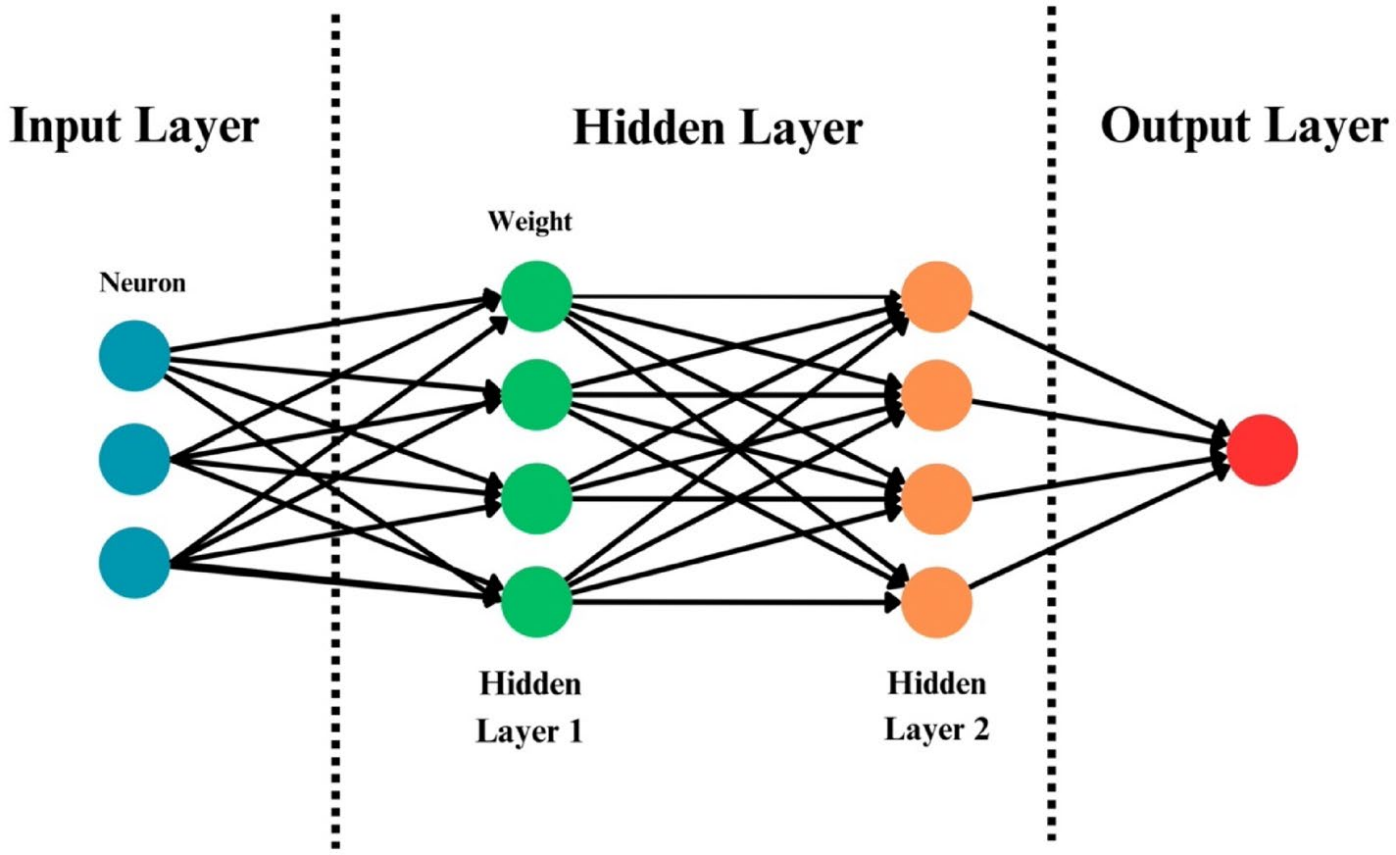
Architecture of SVM.

Support vector machine (SVM) is a supervised learning method for classification and regression, creating hyperplanes in high-dimensional space for distinct separation between classes. It achieves this by maximizing the distance to the nearest training data points, known as the functional margin. This strategic approach ensures enhanced generalization capability of the classifier, underscoring the significance of a larger margin in minimizing the overall prediction error^63^. Figure 4 shows the architecture of support vector machine. Vapnik^64^ Introduced an alternative ε-insensitive loss function to propose ε-support vector regression (SVR)^65^. In essence, SVR aims to identify a function with a maximum deviation of ε from the actual target vectors for the provided training data, while maintaining a flatness criterion^66,67^. The SVR methodology is briefly described as follows: Consider the array vector xi of size n with real value yi. Let F(x) represent a set of real functions, including the regression function f0(x). Addressing the task of approximating the dataset {(x_1,_y_1_),…,(x_n_,y_n_)} with a linear function f(x) = (w⋅x) + b, the optimal regression function is derived by minimizing the empirical risk R as shown in Eq. (5). The details of Kernel functions are shown in Table 2.

**Table 2:** Types of kernel function.

Type of classification	Kernel function
Polynomial degree	$K=\left({x}_{i}.x\right)=[\left({x}^{T}.{x}_{i}\right)+1{]}^{d}$
Gaussian	$K=\left({x}_{i}.x\right)= {e}^{\frac{|\left|x-{x}_{i}\right|{|}^{2}}{{\sigma }^{2}}}$
Multi-layer Perceptron	$K=\left({x}_{i}.x\right)={\text{tanh}}[{(x}^{T}.{x}_{i})]+b$

5 $$R= \frac{1}{n}\sum_{i=1}^{n}{|{y}_{i}-f\left({x}_{i}\right)|}_{\varepsilon }$$

Equation (6) provides ε-insensitive loss function

6 $${\left|y-f\left(x\right)\right|}_{\varepsilon }= \left\{\begin{array}{l}if \left|y-f\left(x\right)\right|\le \varepsilon \\ otherwise \left|y-f\left(x\right)\right|-\varepsilon \end{array}\right.$$

The current objective is to identify a function f(x) that minimizes the deviation, denoted as ε, from the observed target values yi across the entire training dataset. Simultaneously, this function is sought to have a minimal degree of variation, effectively being as flat as possible. Mathematically, this goal translates into the minimization of the following function as shown in Eq. (7).

7 $$R\left(w\right)= \frac{|\left|{w}^{2}\right||}{2}+c\times \frac{1}{n} \sum_{i=1}^{n}|{y}_{i}-f\left({x}_{i}\right){|}_{\varepsilon }$$

where, the first term $\frac{|\left|{w}^{2}\right||}{2}$ considering the flatness of function and the second term $\frac{1}{n} \sum_{i=1}^{n}|{y}_{i}-f\left({x}_{i}\right){|}_{\varepsilon }$. The empirical risk is calculated with the penalty value C, which tunes the trade-off between empirical risk and the flatness of the function. A larger C factor reduces the training error but can lead to decreased generalization performance of the function. Equation (7) can be represented as the dual optimization problem, which can be solved using the Lagrange method as shown in Eq. (8):

8 $${L}_{2}=\sum_{i=1}^{l}{y}_{i}\left({\alpha }_{i}^{*}-{\alpha }_{i}\right)-\varepsilon \times \sum_{i=1}^{l}{y}_{i}\left({\alpha }_{i}^{*}-{\alpha }_{i}\right)-\frac{1}{2} \sum_{i=1}^{l}\sum_{j=1}^{l}\left({\alpha }_{i}^{*}-{\alpha }_{i}\right)\left({\alpha }_{j}^{*}-{\alpha }_{i}\right)({x}_{i}.{x}_{j})$$

Support vectors are the training data points with nonzero Lagrangian multipliers (αi ∗ , αi). The final solution can be expressed as follows:

9 $$f\left(x\right)= \sum_{i=1}^{{n}_{sv}}\left({\alpha }_{i}^{*}-{\alpha }_{i}\right){x}_{i}+b$$

In Eq. (9), ${n}_{sv}$ is the number of support vectors.

### K-nearest neighbors algorithm (KNN)

**Figure 5:**
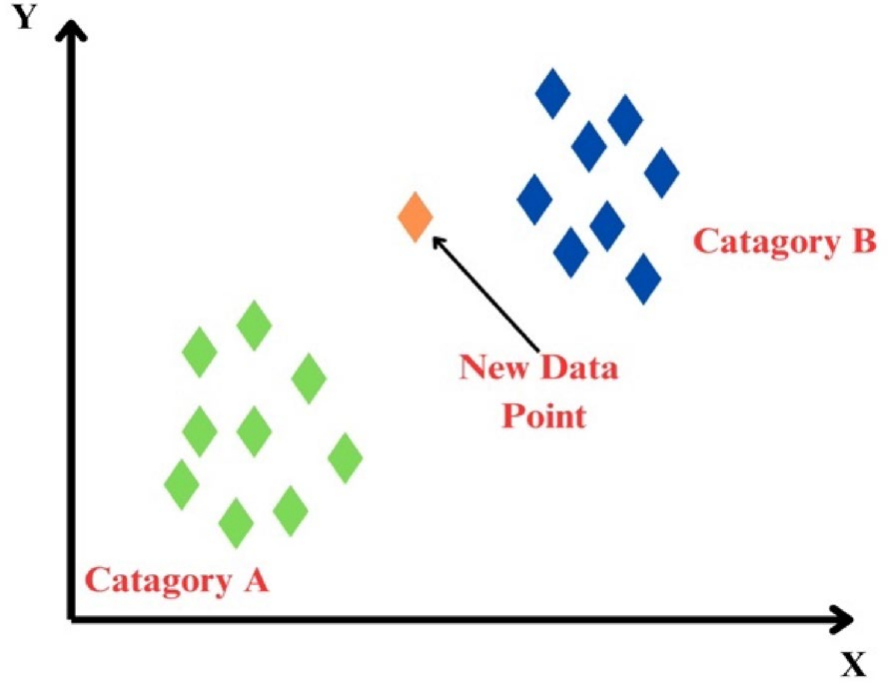
KNN Algorithm.

The KNN approach is a non-parametric that has been used in the early 1970’s in statistical applications^68^. The fundamental principal underpinning k-nearest neighbors (KNN) algorithm involves identifying, within the calibration dataset, a set of k samples that exhibit the closest proximity to the unknown samples, typically determined through distance functions. Subsequently, the classification of these unidentified samples is ascertained by calculating the mean of the response parameters^69^. Consequently, in the context of this classifier, the parameter k assumes a critical role in determining the performance of the k-nearest neighbors (KNN) algorithm. It functions as the primary tuning parameter, profoundly influencing the efficacy of the KNN model^70^. Bootstrap procedure is used to determine parameter k. The graphical presentation of KNN algorithm is illustrated in Fig. 5.

### Artificial neural network (ANN)

**Figure 6:**
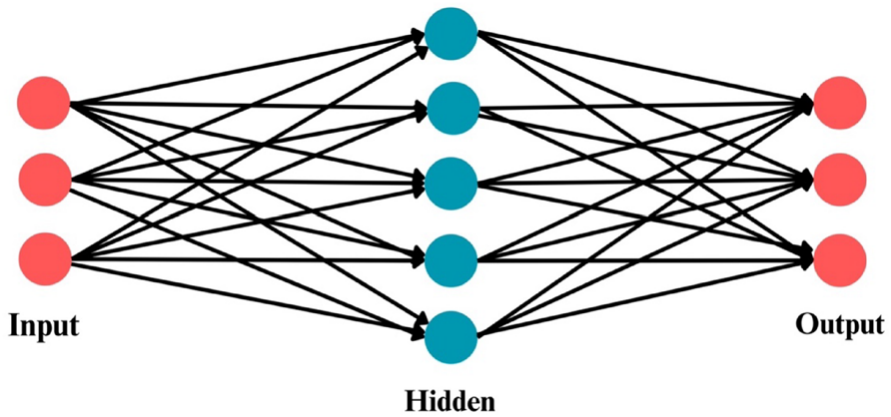
Architecture of artificial neural network.

ANN represents a predictive computational framework derived from the structural principles observed in the human brain. The algorithmic framework of an Artificial Neural Network (ANN) is visually represented in Fig. 6. This network comprises functional units called neurons, interconnected by weights initially set to random values. Throughout the learning process, these weights are adjusted iteratively over multiple epochs, aiming to converge towards an optimal configuration. This iterative adjustment enables the network to develop predictive capabilities with a significant degree of accuracy^71^. In a trained neural network, the desired output is attained through the input signals processed by the network, taking into account the updated weights. The network refines its predictive capabilities by continuously comparing the intended input–output pairs and evaluating the associated errors. The iterative refinement of the machine learning model signifies the potential for enhanced prediction accuracy and establishes the reliability of the predicted outcomes over time.

### Decision tree

**Figure 7:**
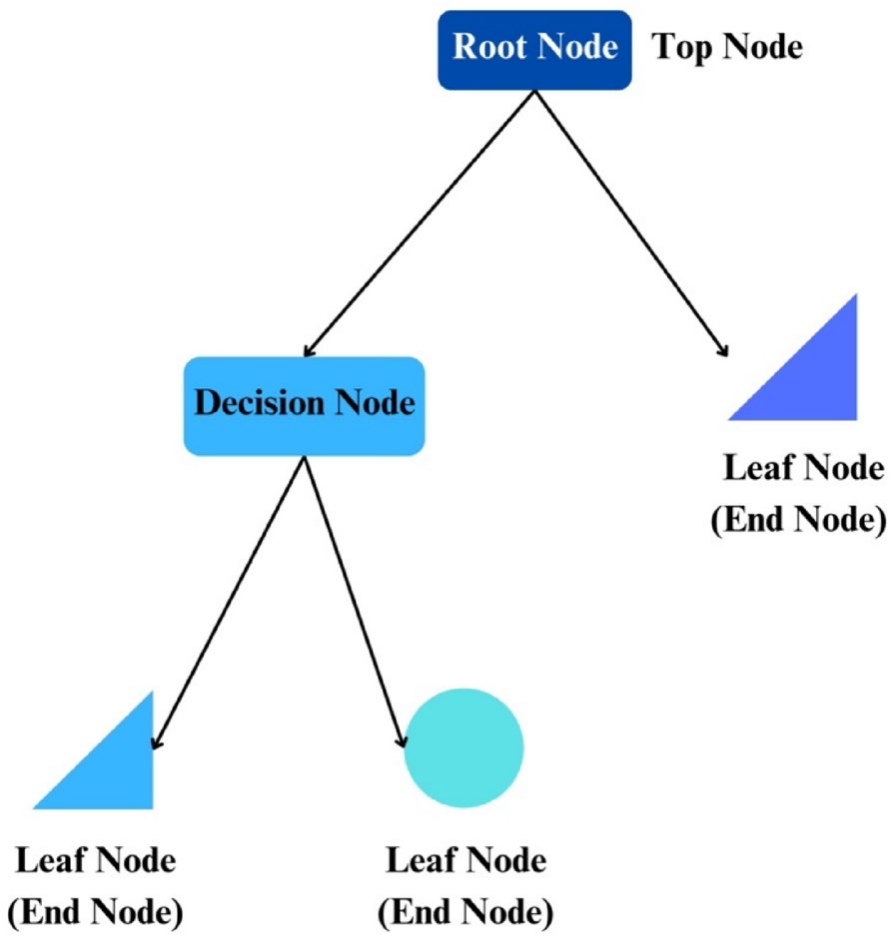
Components of decision tree algorithm.

A decision tree represents a non-parametric supervised learning approach that finds relevance in addressing both classification and regression tasks^72^. Decision trees, characterized by a hierarchical structure with nodes like root, branches, internal nodes, and terminal leaf nodes, classify instances into distinct categories. The process involves recursive testing of features at nodes, creating subtrees based on splitting attributes until no further separation is possible or all instances belong to the same class^73^. In Fig. 7, a comprehensive representation unveils the intricate components that constitute a decision tree. The figure illuminates the structural elements, such as nodes, branches, and leaves, that collectively define the decision-making process within this algorithm.

### Random forest

Random forest regression is proposed by^74^ and is considered an improved classification regression method. Random forest (RF) is distinguished by its notable characteristics, such as speed and adaptability in establishing intricate relationships between input and output functions^75^. Moreover, RF exhibits exceptional efficiency in handling extensive datasets, surpassing other conventional machine learning techniques in this regard. Its versatility has led to its widespread application across diverse domains. For instance, in the banking sector, RF has been instrumental in predicting customer responses, showcasing its practical utility in real-world applications^76^, for predicting the direction of stock market prices^77^, in the medicine/pharmaceutical industry^78^, e-commerce^79^, etc.

The random forest (RF) method encompasses several key steps:

i. Collection of trained regression trees: The process begins with the assembly of trained regression trees employing the provided training set.
ii. Calculation of average output: Subsequently, the average of individual regression tree outputs is computed to obtain a consolidated prediction.
iii. Cross-validation using validation set: The predicted data undergoes cross-validation using a distinct validation set.

**Figure 8:**
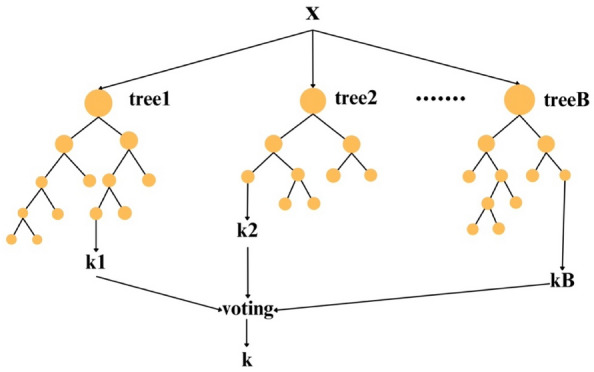
Architecture of random forest.

In this method, a new training set is generated, comprising bootstrap samples derived from the replacement of the original training set. During this phase, certain sample points are substituted with existing ones, while the deleted sample points are preserved in a separate set termed "out-of-bag samples." Approximately two-thirds of the sample points are utilized for estimating the regression function, with the out-of-bag samples serving as the validation set. This process iterates multiple times until the desired accuracy level is attained. A distinctive feature of random forest regression (RFR) lies in its inherent capability to systematically exclude points for out-of-bag samples, subsequently utilizing them for validation purposes. This unique attribute enhances the model's accuracy and reliability. The overall efficacy of each expression tree is determined by calculating the total error at the conclusion of the process, providing valuable insights into the performance of individual trees within the ensemble. In Fig. 8, the architecture of a Random Forest is unveiled, offering a comprehensive view of its structural intricacies.

### AdaBoost

**Figure 9:**
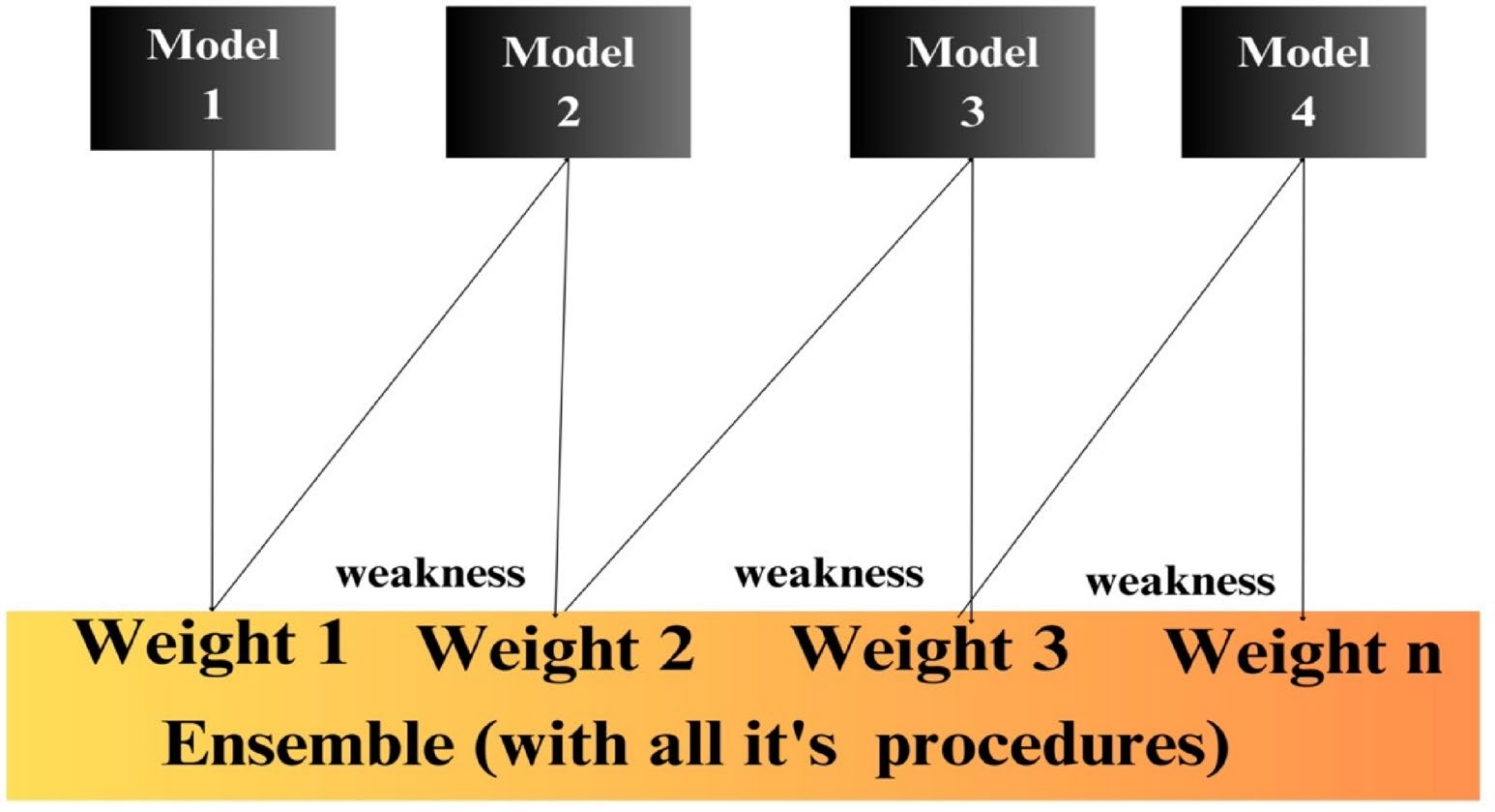
Ada-boost algorithm.

AdaBoost stands as a widely employed boosting algorithm, which systematically constructs an ensemble through multiple iterations. In each iteration, distinct instance weights are applied, and the algorithm adapts to the errors identified by classifiers from preceding iterations^80,81^ adaptively. AdaBoost iteratively adjusts instance weights, focusing on misclassified ones while diminishing emphasis on correct classifications. By combining weak classifiers and utilizing a collective voting mechanism, AdaBoost creates a robust classifier, enhancing performance with each iteration through strengthening weak classifiers and refining the overall classification ability^82^. Figure 9 provides a comprehensive visualization of the AdaBoost algorithm, showcasing the ensemble with its procedural details.

### Voting regressor

**Figure 10:**
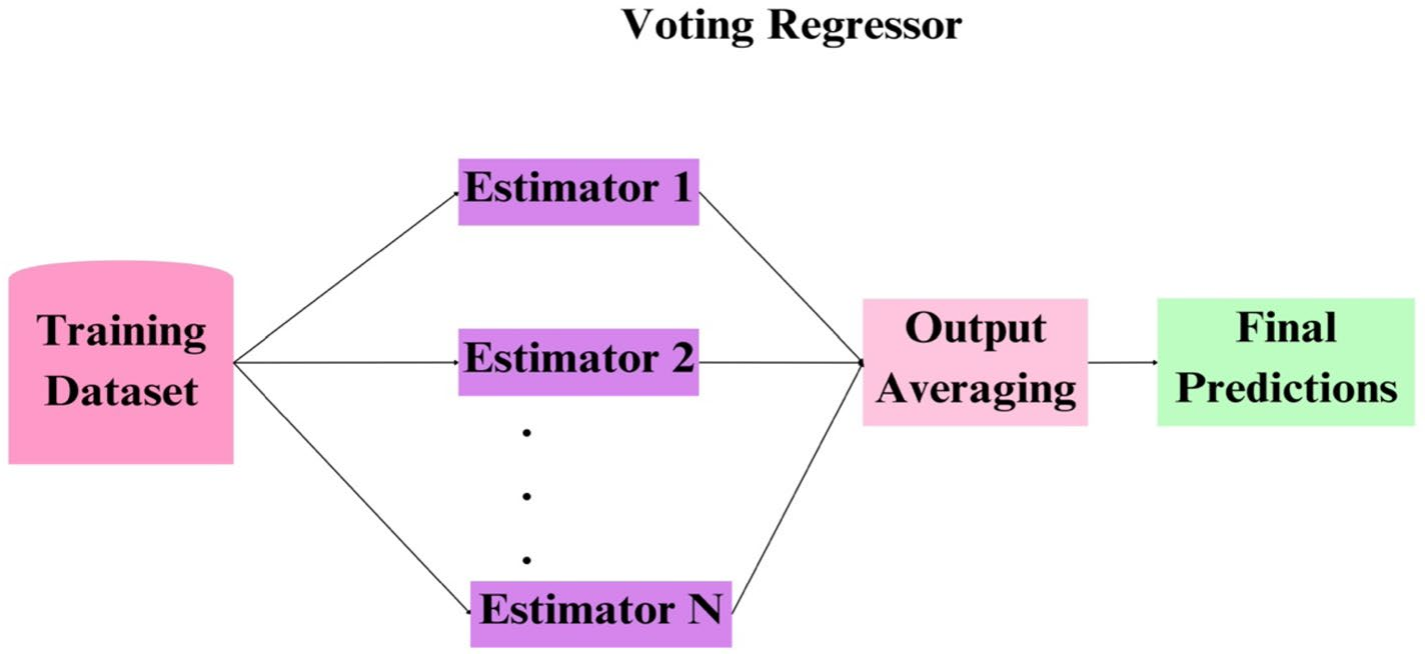
Graphical presentation of Voting Regressor.

The voting regressor, categorized as an ensemble meta-estimator, is a method that sequentially fits multiple base regressors to the entire dataset^83^. The final prediction is generated by averaging multiple estimates. Consequently, the voting mechanism, relying on the performance of multiple models, remains robust against significant errors or inaccuracies from any single model. When one model underperforms, the collective strength of the other models compensates, mitigating the impact of individual model shortcomings. Combining multiple models diminishes the likelihood of a single model generating an erroneous forecast. This approach enhances the robustness of the estimator and reduces susceptibility to overfitting. Figure 10, visually presented a comprehensive view of the sequential stages involved in the Voting Regression process.

### Gradient boost

In 1999, gradient boosting (GB) was introduced as an ensemble strategy specifically designed for regression tasks^84^. Unlike some ensemble methods, GB is exclusively applicable to regression problems. This technique involves evaluating each iteration of a randomly selected training set against the base model. To enhance execution speed and accuracy, practitioners often employ the strategy of randomly subsampling the training data, which not only accelerates the regression process but also aids in averting overfitting.

**Figure 11:**
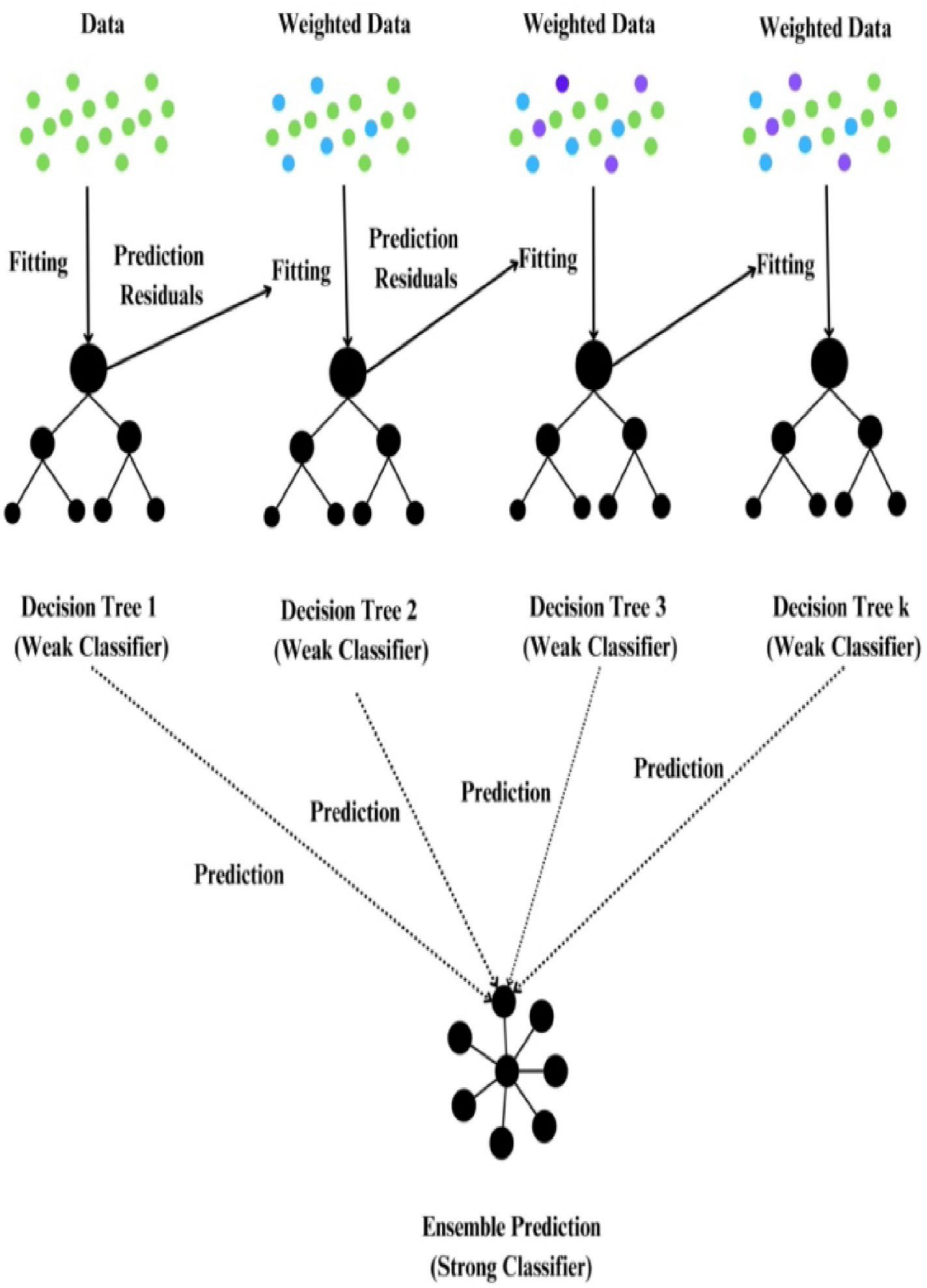
Gradient boost algorithm.

The speed of regression is directly influenced by the proportion of training data utilized, where a smaller percentage necessitates the model to adapt to a reduced dataset during each iteration, thus accelerating the process. However, to ensure optimal performance, careful consideration must be given to tuning parameters. These parameters include 'n-trees,' signifying the number of trees to be generated. Setting 'n-trees' too low can undermine the efficacy of the model. Additionally, the shrinkage factor, commonly known as the learning rate applied to all trees during development, must be calibrated judiciously. Setting the learning rate too high could adversely affect the overall accuracy and stability of the model. Hence, meticulous parameter tuning is imperative to achieve optimal outcomes in Gradient Boosting for regression tasks^85^. The architectural intricacies of gradient boosting are illustrated in Figs. 11 and 13.

### Cat-boost regressor (CBR)

**Figure 12:**
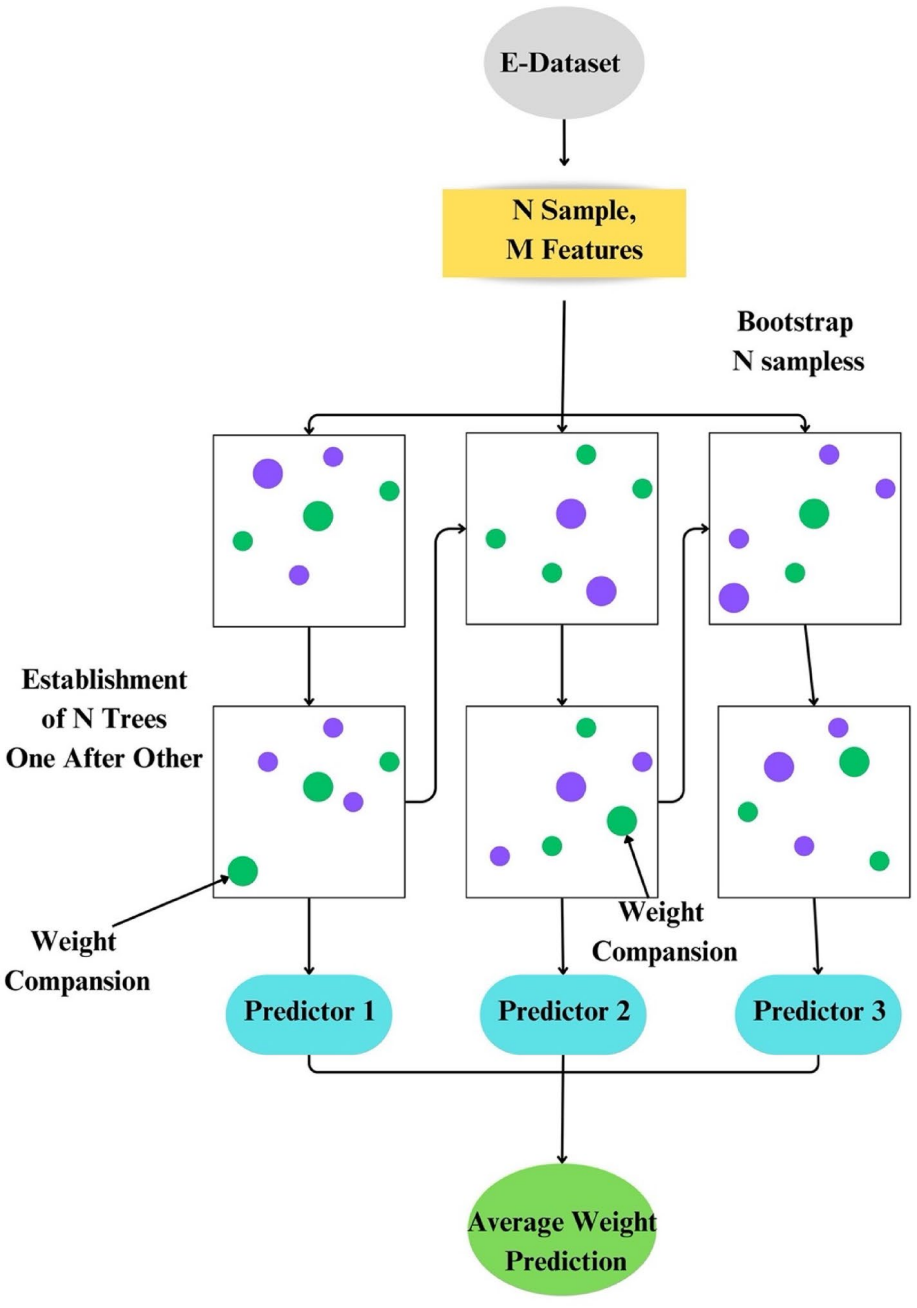
Graphical presentation of Cat-boost algorithm.

Cat Boost, a gradient-boosting decision tree algorithm, stands out for its implementation of balanced trees and handling heterogeneous data. Unlike other boosting methods, it employs symmetric trees and selects feature-split pairs with minimal loss uniformly across nodes. Optimized for efficient CPU use, Cat Boost mitigates overfitting through ordered boosting and computes residuals on separate subsets. While offering a flexible parameter interface, careful hyperparameter consideration is essential for its robustness^86^. In Fig. 12, the architecture of CatBoost, a powerful gradient boosting library, is meticulously illustrated.

### Extreme gradient boosting machine (XG boost)

**Figure 13:**
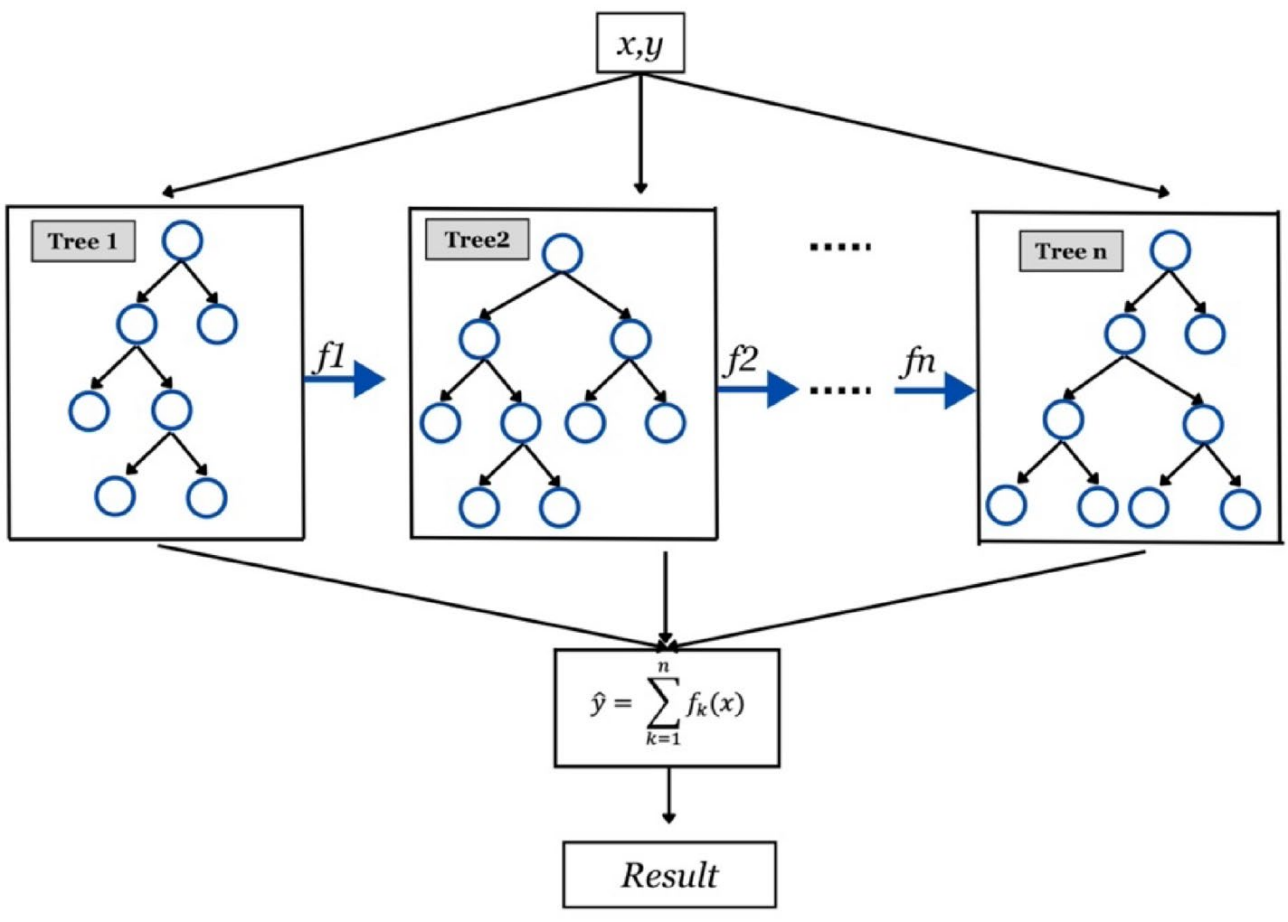
Graphical Presentation of XG-Boost Model.

The XG Boost, proposed by^87^, is enhanced according to the original gradient boosting algorithm^88^. This machine learning approach can be regarded as an ensemble of boosting decision trees, specifically designed to enhance predictive accuracy. Noteworthy is the expeditious model construction phase of XG Boost, facilitated by its parallelizability, allowing for rapid execution^89^. In regression analysis, XG Boost employs the mean squared error loss function, akin to gradient boosting machines (GBM). Throughout the training process, individual regression trees are constructed by fitting them to the residuals of their preceding trees. The construction of these regression trees in the XG Boost model relies on the evaluation of optimal node splits, determined through the utilization of the Similarity Score and Gain index. These metrics play a crucial role in identifying the most effective partitioning points within the decision tree nodes^87^. The Similarity Score is derived from the model residuals and serves as a fundamental metric in XG Boost. Computed from the Similarity Scores of the right leaf, left leaf, and root, the Gain index is pivotal in node selection for regression tree construction. The split with the highest Gain index is chosen to develop the regression tree, thus guiding the model's overall structure. Additionally, the advancement of the construction phase is influenced by the learning rate parameter, which determines the pace at which the model learns. The complexity of each regression tree is modulated by the tree depth parameter, allowing control over the intricacy of the decision boundaries. To mitigate overfitting, a regularization parameter (λ) is integrated into the computation of the Similarity Score, aiding in maintaining model generalization performance during the training phase. The graphical representation of XG-boost model is illustrated in Fig. 13.

## Research methodology

**Figure 14:**
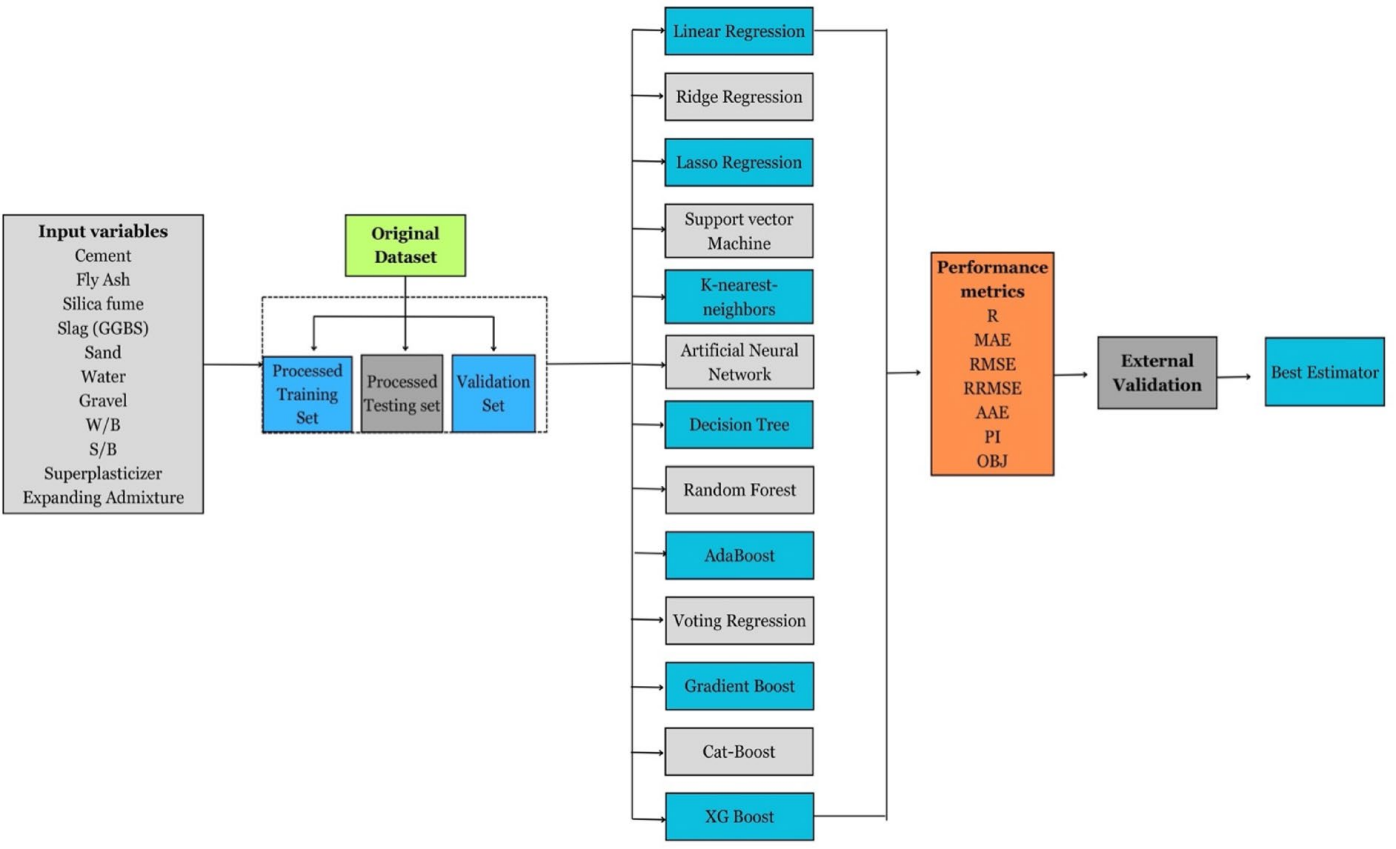
Flow diagram of research methodology.

The flow diagram of research methodology is highlighted in Fig. 14. Initially, the original dataset was partitioned into distinct training (70%), testing (15%) and validation (15%) sets. Subsequently, thirteen distinct machine learning techniques, namely LR, RR, LR, SVM, KNN, ANN, DT, RT, AB, VR, GB, CBR, and XGB were employed. The outcomes generated by these techniques were meticulously compared. Additionally, an external validation analysis was conducted to assess the performance of all machine learning models. Subsequently, the most effective technique that exhibit superior predictive performance was identified and highlighted as the optimal approach in the study.

### Data collection

The dataset used in making predictive models comprises of 261 samples that was taken from the published literature^90^. The specimen set consist of eleven key parameters that have significant influence on the compressive strength of preplaced aggregate concrete. These parameters include cement, fly ash (FA), silica fume (SF), ground granulated blast-furnace slag (GGBFS), sand, water, gravel, water to binder ratio (W/B), sand to binder ratio (S/B), superplasticizer, and expanding admixture. The descriptive analysis of these input variables is presented in Table 3. Measures such as mean, median, and mode were utilized to indicate the central tendency of the data. While extreme values were illustrated through maximum and minimum values. The variability of the data was quantified using standard deviation (SD). A lower SD value suggests that many entities closely align with the mean. Whereas a higher SD indicates a broader distribution range among the entities. Skewness and Kurtosis are utilized to evaluate data symmetry and shape relative to a normal distribution. Positive skewness signifies a rightward tail extension, while negative skewness indicates a leftward tail extension^91^. A skewness value of exactly zero signifies a symmetrical distribution, where the data is evenly spread around the mean without any bias towards the left or right. Skewness values offer valuable insights into the shape and asymmetry of the data distribution. Therefore, helping interpret the underlying characteristics of the variables in the study^92^. A negative kurtosis, denoted as platykurtic, indicates that the data distribution curve is flatter than the normal distribution curve. Conversely, a positive kurtosis, known as leptokurtic, suggests a more peaked curve. A kurtosis value of exactly zero signifies that the data distribution exhibits neither excessive pawedness nor flatness, resembling a normal distribution. These kurtosis values provide crucial insights into the shape and characteristics of the data distribution, aiding in the statistical analysis of the variables under consideration.

**Table 3:** Descriptive analysis of input variables.

Input variables	Unit	Minimum	Maximum	Mean	Std.Daviation	Skewness	Kurtosis
Cement	Kg/m^3^	176	873	390.502	103.320	0.801	1.864
Fly Ash	Kg/m^3^	0	262	11.695	42.508	4.102	17.394
Silica fume	Kg/m^3^	0	57	4.532	11.827	2.652	6.320
Slag (GGBS)	Kg/m^3^	0	228	10.126	40.270	4.045	15.541
Sand	Kg/m^3^	0	873	373.33	176.769	− 0.514	− 0.021
Water	Kg/m^3^	100	431	205.65	60.792	1.248	1.904
Gravel	Kg/m^3^	1.56	2001	1396.453	331.002	− 2.406	8.634
W/B		0.30	0.85	0.5130	0.161	0.793	− 0.490
S/B		0	2	0.975	0.553	0.066	− 0.679
Superplasticizer	Kg/m^3^	0	11.20	3.618	3.697	0.553	− 1.144
Expanding Admixture	Kg/m^3^	0	10.50	1.1029	2.782	2.318	3.863

### Pearson’s correlation analysis

Pearson and Spearman correlation analyses assess relationships between variables. Pearson measures linear correlation strength and direction. While, spearman evaluates monotonic relationships that reveals consistent trends in data patterns^93^. The calculation expression is shown in Eq. (10).

10 $$P(X,Y)= \frac{Cov(X, Y)}{{\sigma }_{x}{\sigma }_{y}}$$

**Figure 15:**
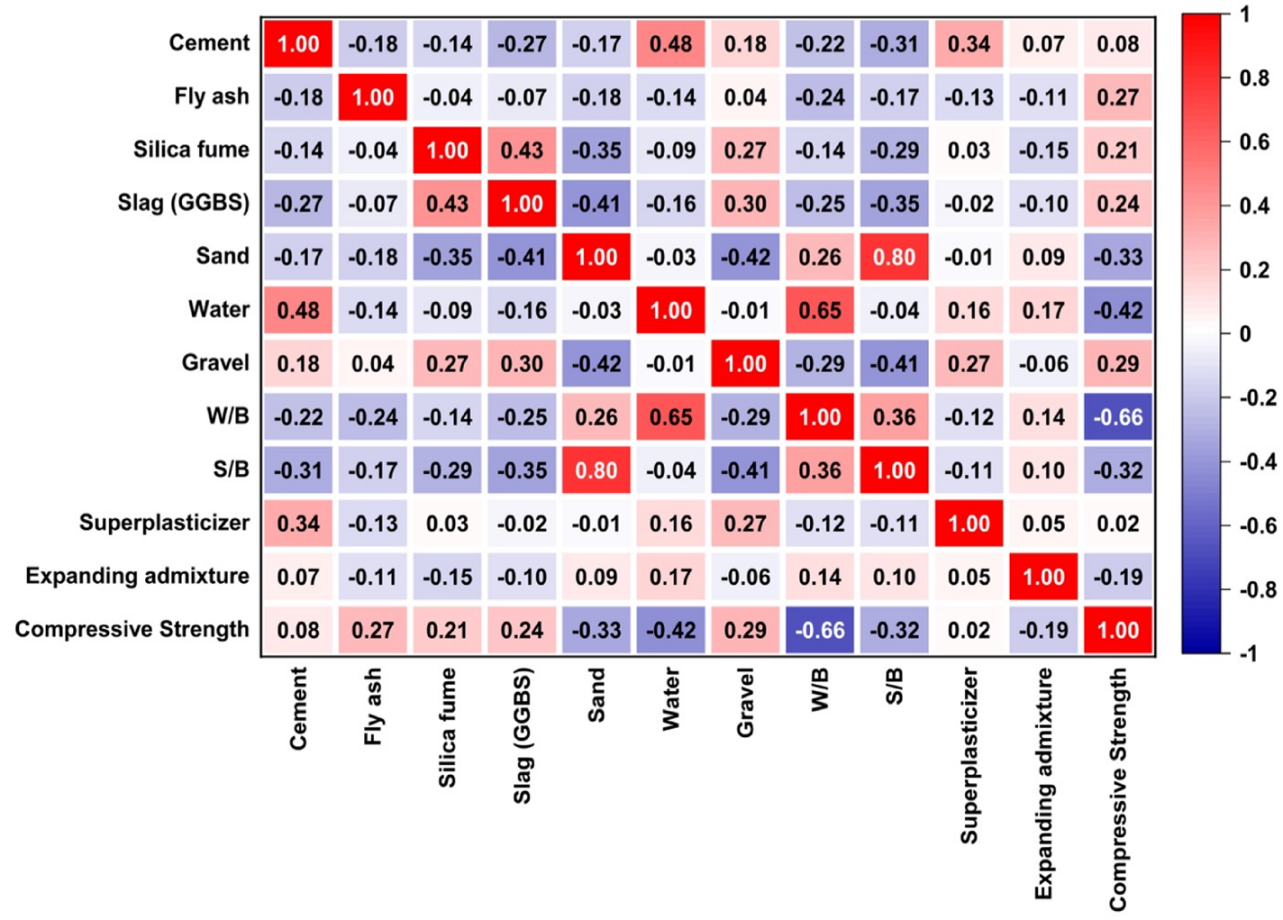
Pearson correlation analysis.

The correlation coefficient (p) measures the strength and direction of the relationship between variables X and Y. A value closer to − 1 or 1 indicates a strong correlation with the sign indicating positive or negative direction. While values near 0 signify a weak correlation. Moreover, the analysis results are presented in Fig. 15. To ensure optimal data analysis and mitigate issues related to multi-collinearity, it is advisable for the correlation coefficient (r) between two input parameters to be below 0.8. Managing multi-collinearity is essential in maintaining the integrity and accuracy of the analytical process^94^. The correlation values unveil the nature and strength of relationships. Cement demonstrates a moderate positive correlation, while fly ash, silica fume, and slag (GGBS) exhibit comparatively stronger positive correlations with compressive strength. Conversely, sand and water reveal moderate to relatively strong negative correlations. Thus, indicating an inverse relationship with compressive strength. Gravel showcases a moderate positive correlation. Whereas water to binder ratio (W/B) and sand to binder ratio (S/B) depict robust negative correlations. Superplasticizer demonstrates a minute positive correlation, and expanding admixture displays a weak negative correlation with strength. These correlations elucidate the extent and directionality of the linear interplay between each input variable and compressive Strength. Notably, variables like water and W/B ratio exhibit robust negative correlations, suggesting an inverse relationship with concrete strength. Conversely, variables like fly ash and silica fume display moderate positive correlations, signifying a direct relationship with compressive strength.

### Model development

In the realm of machine learning models, there exist two distinct types of parameters. First are the model parameters, gleaned directly from the data itself. The second category encompasses hyperparameters, predetermined prior to the initiation of the training process. The effective optimization of hyperparameters is a pivotal element in attaining optimal model performance^95,96^. The determination of hyperparameter values for the machine learning models in the investigation involved a meticulous and comprehensive approach. Initial selections were guided by insights from a thorough literature review, incorporating established best practices within the field. Additionally, a systematic trial-and-error process was implemented to iteratively refine hyperparameter values through experiments. Thus, enabling the observation and analysis of their impacts on model performance. Thirteen distinct machine learning models were employed to identify the optimal predictive model for the compressive strength of PAC. The algorithms utilized encompassed linear regression, voting regressor, lasso regression, random forest, decision tree, support vector machine, K-nearest neighbors, artificial neural network, cat boost, ada-boost, ridge regression, XG boost, and gradient boost. A comprehensive overview of these machine learning models, along with their respective parameters and values is presented below for detailed analysis (Table 4).

**Table 4:** Hyper-parameters values of the proposed machine learning models.

Model	Parameter	Values considered	Optimal
LR	Normalization	True, False	True
RR	Alpha	[0.01, 10, 100, 1000]	0.01
	Fit intercept	True, False	True
	solver	'auto', 'svd', 'cholesky', 'lsqr', 'sparse_cg', 'sag', 'saga'	'auto'
Lasso	Alpha	[0.01, 0.1, 1, 10, 100]	0.1
	Fit intercept	True, False	True
DT	Max Depth	[None, 5, 10, 20, 30]	10
	Criterion	'gini', 'entropy','mse', 'mae'	'mse'
RF	The number of trees in the forest	Integer (e.g., 50, 100, 200, etc.)	100
	Minimum Sample for Split	[2, 5, 10]	2
	Minimum samples of leaf node	[1, 2, 4]	1
	Criterion	'gini', 'entropy','mse', 'mae'	'mse'
SVR	Regularization parameter, C	Positive float	1
	Kernel	'linear', 'poly', 'rbf', 'sigmoid'	'rbf'
	Kernel coefficient, gamma	Auto, scale, or a positive float	'scale'
ANN	Activation	'identity', 'logistic', 'tanh', 'relu'	'relu'
	Alpha	[0.0001, 0.001, 0.01]	0.0001
	Hidden Layer Size	[(50, 50), (100,), (50, 100, 50)]	(100, 50)
	Learning Rate	[0.0001, 0.001, 0.01]	0.001
	Maximum Iteration	[100, 200, 300,…]	1000
	solver	'lbfgs', 'sgd', 'adam'	'adam'
KNN	Algorithm	'auto', 'ball_tree', 'kd_tree', 'brute'	'auto'
	Number of neighbors	Positive Integer	5
	Power parameter, p	[1, 2]	2
	Leaf Size	Positive Integer	30
	Weight function	'uniform', 'distance'	'uniform'
AB	Learning Rate	[0.01, 0.1, 0.5]	0.1
	The loss function	'linear','exponential','square':	'linear'
	The maximum number of estimators	[50, 100, 200, …]	500
CBR	Bagging Temperature	[0, 1]	1
	Depth	[4, 6, 8, …]	6
	Eval Metric	'Logloss','RMSE'	'RMSE'
	Iterations	[100, 200, 300, …]	100
	Learning Rate	[0.01, 0.1, 0.2, …]	0.05
	Metric Period	[10, 20, 50, …]	10
	Od Type	'Iter','IncToDec'	'Iter'
	Od Wait	[10, 20, 50, …]	20
	Random Seed	Integer	42
	Subsample	[0.5, 0.8, 1.0]	0.8
VR	CatBoost Regressor Weight	Integer	0.2
GB	Learning Rate	[0.01, 0.1, 0.2, …]	0.1
	The loss function	'ls', 'lad', 'huber', 'quantile','deviance'	'ls'
	Maximum Depth	[3, 5, 7, …]	5
	The number of features	'auto', 'sqrt', 'log2', None	'auto'
	The maximum number of estimators	[50, 100, 200, …]	200
	Minimum samples of leaf node	[1, 2, 5, …]	1
XGB	Subsample ratio of the training	[0.1–1]	0.95
	Col sample by Tree	[0.1–1]	0.75
	Learning Rate	[0.01, 0.1, 0.2, …]	0.05
	Max Depth	[3, 5, 7, …]	7
	Minimum Child Weight	[1, 3, 5]	5
	The maximum number of estimators	[50, 100, 200, …]	200

### Evaluating model performance using statistical metrics

Various regression error metrics were employed including accuracy, scale-dependent metrics, and percentage-error metrics in evaluating machine learning models. The selection of specific metrics depended on the model type and implementation strategy. Key evaluation metrics included correlation coefficient (r), mean absolute error (MAE), root mean square error (RMSE), relative root mean square error (RRMSE), performance index (PI), and objective function (OBJ). Equation (11–16) represent these key evaluation metrics, condensing residuals into single values to represent model predictive capability for large datasets.

11 $$R= \frac{\sum_{i=1}^{n}\left(a{c}_{i}- \overline{a{c }_{i}}\right)({pr}_{i}-\overline{\overline{{pr}_{i}}})}{\sqrt{\sum_{i=1}^{n}{\left(a{c}_{i}- \overline{a{c }_{i}}\right)}^{2}{({pr}_{i}-\overline{\overline{{pr}_{i}}})}^{2}}}$$

12 $$MAE= \frac{\sum_{i=1}^{n}|{ac}_{i}- {pr}_{i}|}{n}$$

13 $$RMSE= \sqrt{\frac{\sum_{i=1}^{n}{({ac}_{i}- {pr}_{i})}^{2}}{n}}$$

14 $$RRMSE= \frac{1}{|\stackrel{-}{ac|}} \sqrt{\frac{\sum_{i=1}^{n}{({ac}_{i}- {pr}_{i})}^{2}}{n}}$$

15 $$PI= \frac{RRMSE}{1+R}$$

16 $$OBJ=\left(\frac{{No}_{train}-{No}_{test}}{{No}_{all}}\right)\frac{{MAE}_{train}}{{r}_{train}^{2}}+\frac{{2No}_{test}}{{No}_{all}} \times \frac{{MAE}_{test}}{{r}_{test}^{2}}$$

The study evaluates the predictive accuracy of various models for compressive strength (CS) in PAC. Correlation coefficient (R) measures the relationship between actual and predicted values in which R > 0.8 indicates strong accuracy. R2 gauges model variability utilization, with values near 1 indicating effective parameter usage^97^. Root mean square error (RMSE) reflects prediction errors^98^, while mean absolute error (MAE) handles continuous datasets efficiently^99^. Performance Index (PI) values near 0 indicate strong model performance. External validation confirms the accuracy of the models. Some differences were noticed, but they align with specific criteria and are similar to findings in previous research^100–102^. The details of different parameters for external validation is shown in Table 5.

**Table 5:** Parameters for external validation.

Sr.No.	Equation	Range
1	R	0.8 < R
2	$k= \sum_{i=1}^{n}\frac{({e}_{i}\times {p}_{i})}{{e}_{i}^{2}}$	0.85 < *k* < 1.15
3	${k}^{2}=\sum_{i=1}^{n}\frac{({e}_{i}\times {p}_{i})}{{p}_{i}^{2}}$	0.85 < *k* < 1.15
4	$m= \frac{{R}^{2}-{R}_{o}^{2}}{{R}^{2}}$	m < 1
5	$n= \frac{{R}^{2}-{R}_{o}^{{\prime}2}}{{R}^{2}}$	n < 1
6	${R}_{m}= {R}^{2}(1-\sqrt{|{R}^{2}-{R}_{o}^{2}|}$	0.5 < ${R}_{m}$
7	${R}_{o}^{2}=1-\frac{\sum_{i=1}^{n}({p}_{i}-{e}_{i}^{o}{)}^{2}}{\sum_{i=1}^{n}({p}_{i}-{p}_{i}^{o}{)}^{2}}$	${R}_{o}^{2}\cong 1$
8	${R}_{o}^{{\prime}2}=1-\frac{\sum_{i=1}^{n}({e}_{i}-{p}_{i}^{o}{)}^{2}}{\sum_{i=1}^{n}({e}_{i}-{e}_{i}^{o}{)}^{2}}$	${R}_{o}^{2}\cong 1$
	${e}_{i}^{o}=k\times {p}_{i}$ ${p}_{i}^{o}=k\times {e}_{i}$	

## Results and discussion

### Comparison of regression plots

**Figure 16:**
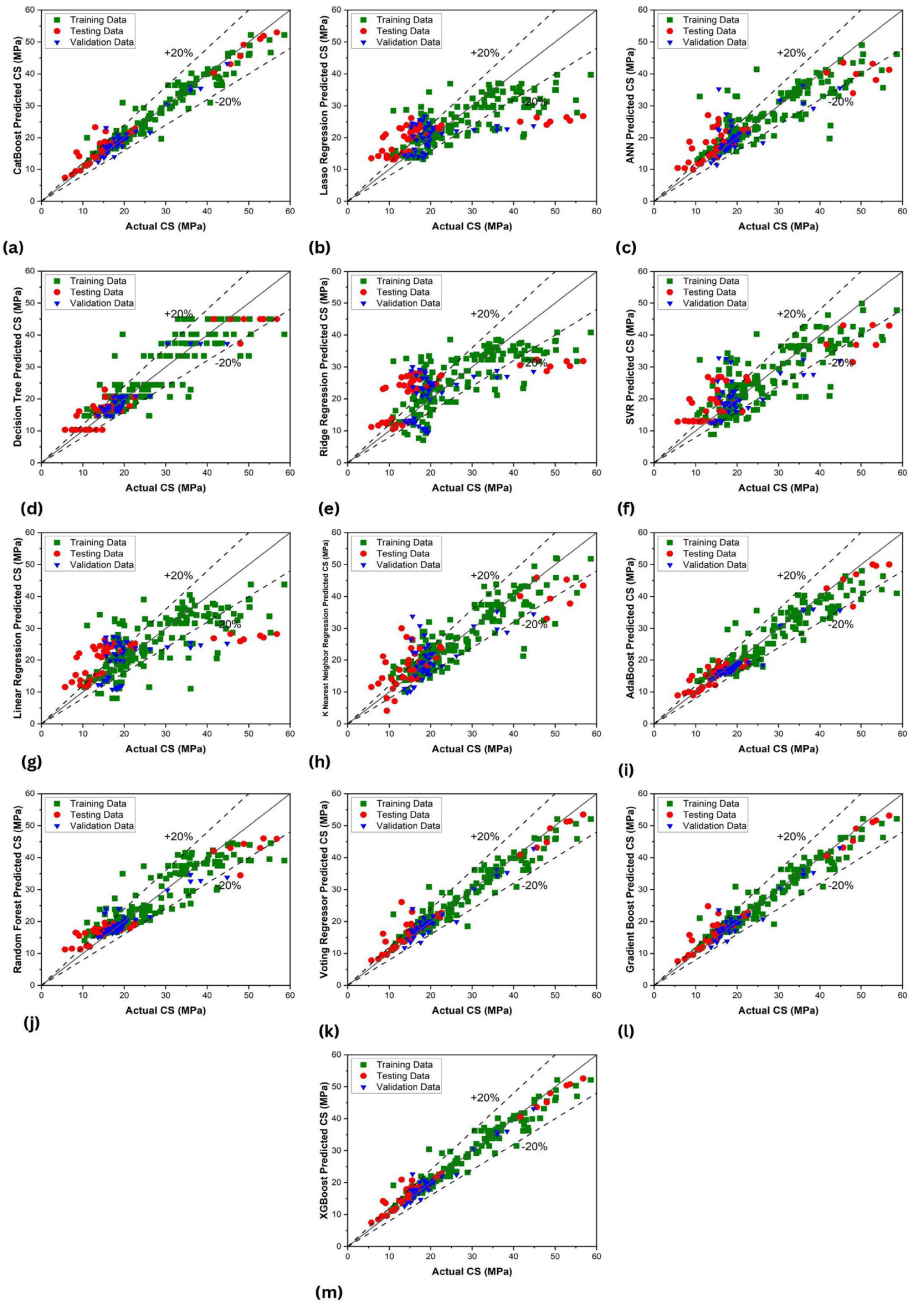
Scatter plots of the predicted compressive strength of PAC based on ML models and input features; (**a**) cat-boost approach; (**b**) lasso regression; (**c**) ANN; (**d**) decision tree; (**e**) ridge regression; (**f**) SVR; (**g**) linear regression; (**h**) KNN; (**i**) Adaboost; (**j**) random forest; (**k**) voting regressor; (**l**) gradient boost; (**m**) XGboost.

Figure 16 presents the results of the predicted compressive strength of PAC based on ML models. The predicted training data points were alongside the X = Y line, which shows XG Boost has the highest R^2^ value among all the models, which means surpassing other methods in predictive accuracy. This score suggests it effectively captures the underlying data patterns, making it the apparent frontrunner based on the given R^2^ value. Following closely is CatBoost with an impressive R^2^ of 0.9512, demonstrating similar robustness in prediction capability. Gradient Boosting also proves its efficacy, achieving an R^2^ of 0.9461. The Voting Regressor, leveraging predictions from multiple models, delivers a commendable R^2^ of 0.9401, indicating a strong fit to the data. AdaBoost and Random Forest showcase satisfactory performance with R^2^ values of 0.908 and 0.8524, respectively, while Decision Tree achieves a respectable R^2^ of 0.8197, placing it among the better performing models. Compared to these top performers, ANN, KNN, and SVM exhibit moderate fits with R^2^ values of 0.7654, 0.7602, and 0.6689, respectively. Lastly, Lasso and Ridge Regressor display relatively weaker fits with R^2^ values of 0.5230 and 0.4902, respectively. This indicates their limited explanatory power in this specific context.

The variations in performance metrics across the diverse set of machine learning models can be attributed to specific characteristics inherent in each model. Commencing with the Cat Boost (CB) model, its outstanding performance in the training subset as reflected by a correlation coefficient (R) of 0.9700, low mean absolute error (MAE), and Root Mean Squared Error (RMSE) can be ascribed to its inherent ability to handle categorical features efficiently. The model's robustness in capturing complex patterns and relationships contributes to its superior performance in the testing and validation subsets. In contrast, the Lasso Regression Model with a regularization approach exhibited lower R values in both the training and testing subsets. This indicates a tendency to favour sparsity in the model, leading to a weaker linear relationship. The higher MAE and RMSE values can be attributed to the regularization penalty, which may result in larger prediction errors and sensitivity to outliers. The artificial neural network (ANN) model's balanced performance across subsets can be linked to its capacity to capture intricate patterns. The ANN model excels in learning hierarchical representations of data, contributing to its competitive performance in both testing and validation subsets. The decision tree (DT) model's robustness in capturing complex patterns and relationships is evident in its high R values and low MAE and RMSE scores across subsets. The non-linear nature of decision trees allows them to adapt well to the training data, resulting in accurate predictions in testing and validation subsets. Ridge regression (RR), characterized by a regularization approach, exhibited a trade-off between bias and variance. While the model demonstrated lower R values in the training set, its ability to generalize well in testing and validation subsets is reflected in the lower MAE and RMSE values. The Support Vector Machine (SVM) model showcased exceptional performance in the training subset, marked by a high correlation coefficient and an impressive Performance Index. However, this high performance may indicate overfitting, as evidenced by the relatively lower performance in the testing and validation subsets. SVM's susceptibility to outliers might contribute to this observed variance. Linear regression (LR), being a simpler model relying on linear relationships, demonstrated moderate performance across subsets. The consistent R values indicate a reliance on linear patterns, while the higher MAE and RMSE scores highlight limitations in capturing more intricate relationships present in the data. The K-nearest neighbors (KNN) model, which relies on proximity-based predictions, displayed strong correlations and lower prediction errors in the training subset. However, its performance diminished in testing and validation subsets, indicating challenges in generalization and potential sensitivity to noise. For the AdaBoost (AB) model, the high R values and low errors in the training set can be attributed to the model's adaptability to weak learners. However, the observed drop in performance in the testing and validation subsets suggests potential overfitting or limitations in capturing diverse patterns. The random forest (RF) model demonstrated consistent performance metrics across subsets. Its ensemble approach, combining multiple decision trees, allows for robust pattern recognition, leading to stable performance in both training and testing subsets. The voting regressor (VR) model's robust performance across subsets can be linked to its ability to aggregate predictions from multiple base models. This ensemble approach contributes to stable performance, as seen in the competitive R values and low errors across subsets. The Gradient Boosting (GB) and XGBoost (XGB) models exhibited consistent performance metrics, leveraging the boosting technique to sequentially improve model accuracy. These models, characterized by high R values and low errors, demonstrate their effectiveness in capturing complex patterns across subsets. In summary, the unique characteristics of each machine learning model contribute to the observed variations in performance metrics across subsets. Models with the ability to capture complex patterns, handle categorical features efficiently, and adapt well to diverse datasets exhibit superior performance, while simpler models may demonstrate moderate performance with limitations in capturing intricate relationships (Table 6).

**Table 6:** Description of each model’s performance metrics in the training and testing datasets.

Model	Subset	R	MAE	RMSE	RRMSE	PI	OBJ
CB	Training	0.9700	1.6872	2.6760	0.1000	0.0507	1.5840
	Testing	0.9860	1.7093	2.7695	0.1380	0.0694	
	Validation	0.9640	1.1034	1.8326	0.0893	0.0455	
LR	Training	0.7624	4.9981	7.3414	0.2745	0.1557	15.862
	Testing	0.6625	8.8728	11.8150	0.5887	0.3541	
	Validation	0.3659	4.6138	6.5105	0.3174	0.2324	
ANN	Training	0.8626	3.3507	5.4703	0.2045	0.1098	4.7093
	Testing	0.9247	4.4382	6.4245	0.3201	0.1663	
	Validation	0.7173	3.0537	4.9429	0.2410	0.1403	
DT	Training	0.8729	3.6410	5.2245	0.1953	0.1043	3.5365
	Testing	0.9636	3.3062	4.3683	0.2176	0.1108	
	Validation	0.9400	1.7655	2.3507	0.1146	0.0590	
RR	Training	0.7291	5.4395	7.3219	0.2737	0.1583	18.1793
	Testing	0.6745	8.9614	11.0077	0.5485	0.3275	
	Validation	0.4040	6.2531	7.4291	0.3622	0.2580	
SVM	Training	0.8137	4.5445	6.2574	0.2339	0.1290	7.5656
	Testing	0.8885	6.1296	7.6325	0.3803	0.2013	
	Validation	0.5577	4.2681	6.0484	0.2949	0.1893	
LR	Training	0.7165	5.3528	7.5736	0.2832	0.1649	14.766
	Testing	0.6609	8.9493	11.4833	0.5722	0.3445	
	Validation	0.3747	5.7745	7.0526	0.3439	0.2501	
KNN	Training	0.8850	3.5525	4.9821	0.1863	0.0988	6.0977
	Testing	0.8800	5.2991	7.1608	0.3568	0.1897	
	Validation	0.3657	4.2954	5.7836	0.2820	0.1724	
AB	Training	0.9360	2.7500	4.0245	0.1504	0.0777	2.4066
	Testing	0.9812	2.2034	3.1302	0.1559	0.0787	
	Validation	0.9628	1.4682	2.3173	0.1130	0.0575	
RF	Training	0.9017	3.2521	4.6617	0.1743	0.0916	3.4306
	Testing	0.9719	3.7412	4.8040	0.2393	0.1214	
	Validation	0.9104	1.9033	3.0584	0.1491	0.0780	
VR	Training	0.9650	1.8676	2.8851	0.1078	0.0549	1.8040
	Testing	0.9801	1.8526	3.1855	0.1587	0.0801	
	Validation	0.9488	1.3446	2.1691	0.1057	0.0542	
GB	Training	0.9678	1.7720	2.7687	0.1035	0.0526	1.6888
	Testing	0.9830	1.7848	2.9853	0.1487	0.0750	
	Validation	0.9566	1.2158	2.0041	0.0977	0.0499	
XGB	Training	0.9735	1.5860	2.5417	0.0950	0.0481	1.4987
	Testing	0.9910	1.6454	2.4333	0.1212	0.0609	
	Validation	0.9688	1.0730	1.7142	0.0835	0.0424	

### Models error assessment

**Figure 17:**
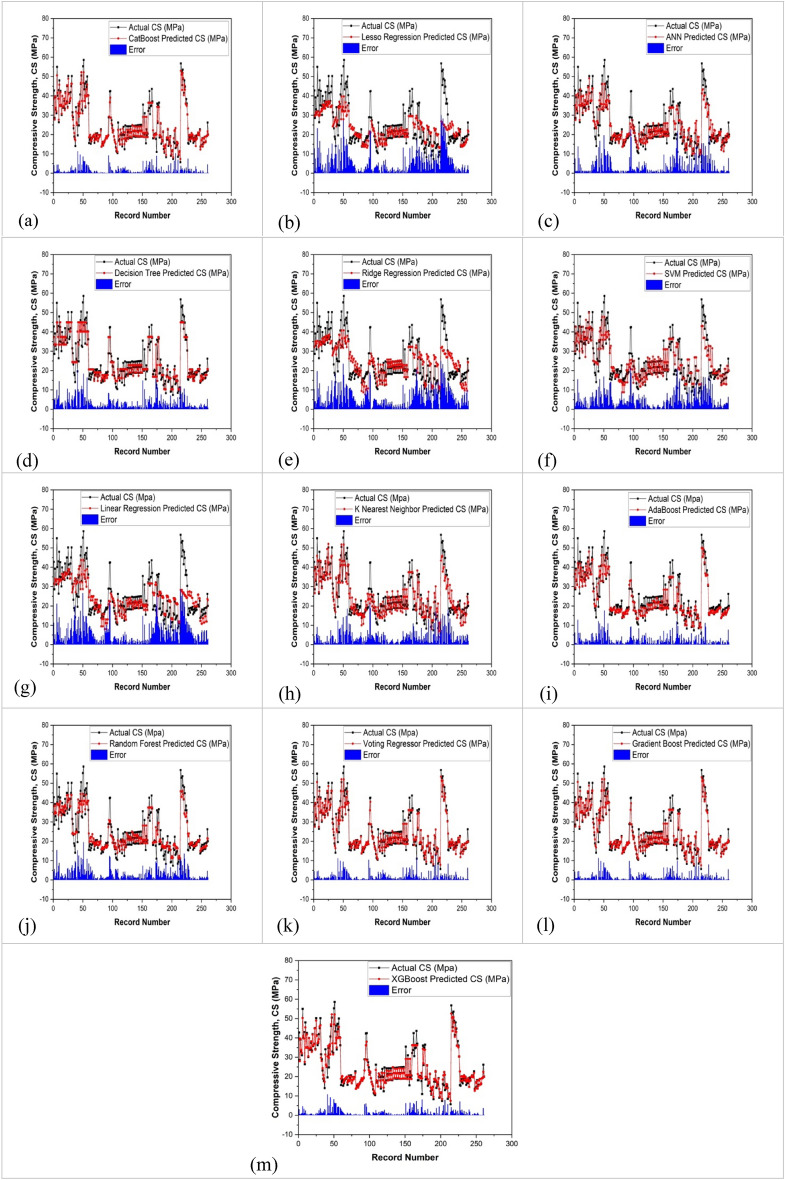
Description of the absolute error among model predicted and actual data for Compressive Strength (CS); (**a**) cat-boost approach; (**b**) lasso regression; (**c**) ANN; (**d**) decision tree; (**e**) ridge regression; (**f**) SVR; (**g**) linear regression; (**h**) KNN; (i) Adaboost; (**j**) random forest; (**k**) voting regressor; (**l**) gradient boost; (**m**) XGboost.

In the assessment of the models' predictions for the compressive strength of preplaced aggregate concrete, Fig. 5 was generated to offer a visual representation of the error metrics, providing a detailed analysis of the model's performance. This figure serves as a pivotal tool for comprehending the accuracy of the predictions in relation to the actual data. The mean absolute error (MAE) values obtained from diverse models, including Cat Boost (CB), linear regression (LR), artificial neural network (ANN), decision tree (DT), ridge regression (RR), support vector machine (SVM), K-nearest neighbours (KNN), AdaBoost (AB), random forest (RF), voting regressor (VR), gradient boosting (GB), and XG Boost (XGB), were calculated and compared. The statistical assessment across thirteen models shed light on their respective predictive capabilities. Cat-boost exhibited notable consistency with a relatively low mean and median error of 1.61 and 0.86, respectively, suggesting stable performance along with moderate variance and standard deviation. In contrast, Lasso and ANN displayed higher mean and median errors (5.55/3.37 and 4.26/2.49, respectively) compared to Cat-boost, indicating less accuracy and considerable variability. Decision Tree showcased commendable predictive ability, demonstrating a low mean and median error (1.47/0.70) with moderate variance. However, models like Ridge, SVM, and KNN revealed moderate performance and variability. Notably, LR stood out with relatively low mean and median errors (1.16/0.51), indicating better performance than several other models. Gradient Boosting presented promising results with lower mean error (1.31), suggesting relatively better predictive performance, while XG Boost showed moderate mean error (1.65) but stable performance. Overall, models displaying lower mean/median errors and stable variance, such as Cat-boost, Decision Tree, LR, Gradient Boosting, and XG Boost, showcased comparatively better performance in predicting the compressive strength of preplaced aggregate concrete. These results offer valuable insights into the strengths and weaknesses of each model, facilitating well-informed decisions regarding their practical application in predicting the compressive strength of preplaced aggregate concrete. In Fig. 17, a detailed portrayal is presented, illustrating the absolute error between the predicted and actual data for compressive strength (CS).

### Sensitivity analysis

**Figure 18:**
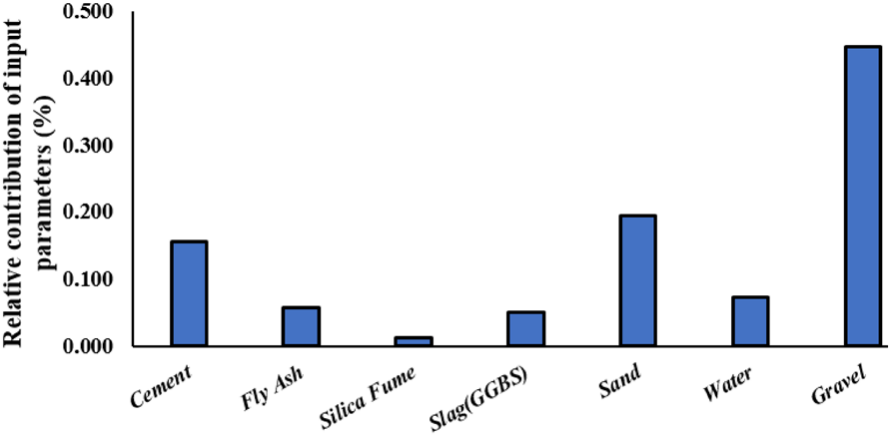
Sensitivity Analysis.

The sensitivity analysis bar chart serves as a visual representation elucidating the relative contributions of distinct input parameters to the overall sensitivity of the model. Positioned on the y-axis, the proportions or percentages of sensitivity associated with each input parameter are depicted, while the x-axis meticulously outlines the corresponding input variables. This comprehensive examination delves into the nuanced insights provided by the sensitivity analysis bar chart Fig. 18. Cement, registering a sensitivity contribution of 15.6%, emerges as a pivotal factor significantly influencing the model's output. Perturbations in cement content wield a moderate yet impactful effect on the overall sensitivity of the model. Fly Ash, with a sensitivity contribution of 5.9%, exhibits a comparatively lower but still noteworthy influence on the model's output. Its impact, while less conspicuous than cement, remains a pertinent factor shaping the model's sensitivity landscape. Silica Fume, with a sensitivity contribution of 1.3%, manifests a diminished impact relative to cement and fly ash. Although its influence is relatively minor, silica fume plays a discernible role in shaping the model's sensitivity dynamics. Ground Granulated Blast-furnace Slag (GGBS), contributing 5.1% to sensitivity, occupies a moderate position among the input parameters. Variations in slag content exert a notable effect on the overall sensitivity of the model. Sand stands out as a highly sensitive input parameter, boasting a substantial contribution of 19.5%. This prominence signifies that fluctuations in sand content wield a profound influence on the model's sensitivity and subsequent output. Water, with a sensitivity contribution of 7.4%, underscores its significance in influencing the model, albeit not as dominantly as sand or gravel. Changes in water content exert a substantial effect on the model's sensitivity dynamics. Gravel, the most sensitive input parameter, commands a significant contribution of 44.7%. This heightened sensitivity underscores the pivotal role of gravel in shaping the overall sensitivity of the model.

### External validation

The results from the external validation Table 7 provide a detailed insight into the performance of different models in predicting the compressive strength of preplaced aggregate concrete. The table presents various metrics such as correlation coefficient (r), slope (k), normalized slope (k'), coefficient of determination ${R}_{o}^{2}$, normalized coefficient of determination ${R}_{o}^{\mathrm{^{\prime}}2}$, Nash–Sutcliffe Efficiency (Rm), Willmott's index (m), and index of agreement (n) for each model. These metrics collectively provide a comprehensive overview of each model's ability to accurately predict the compressive strength of preplaced aggregate concrete. Researchers can use this information to make informed decisions about selecting the most suitable model based on the specific requirements of their study. The models with higher correlation coefficients, better explanatory power (${R}_{o}^{\mathrm{^{\prime}}2}$), and superior predictive accuracy (higher Rm and positive 'm') are particularly noteworthy in the context of this research. Cat Boost (CB) demonstrates high correlation (r = 0.9753) and normalized coefficient of determination (${R}_{o}^{\mathrm{^{\prime}}2}$=0.9631), indicating strong predictive power. The model's performance metrics such as Nash–Sutcliffe Efficiency (Rm = 0.9364) and Willmott's index (m = 0.0317) also show good accuracy. Linear Regression (LR) exhibits moderate correlation (r = 0.7232) and normalized coefficient of determination (${R}_{o}^{\mathrm{^{\prime}}2}$=0.8233). However, the model's Nash–Sutcliffe Efficiency (Rm = 0.5137) and Willmott's index (m = − 0.0500) indicate a less accurate fit to the data. Artificial Neural Network (ANN) shows a high correlation (r = 0.8749) and normalized coefficient of determination (${R}_{o}^{\mathrm{^{\prime}}2}$=0.9245). The Nash–Sutcliffe Efficiency (Rm = 0.7356) and Willmott's index (m = − 0.0912) suggest reasonably good predictive accuracy. Decision Tree (DT) demonstrates strong correlation (r = 0.9054) and normalized coefficient of determination (${R}_{o}^{\mathrm{^{\prime}}2}$=0.9701), indicating excellent predictive power. The Nash–Sutcliffe Efficiency (Rm = 0.7674) and Willmott's index (m = − 0.1460) further support its accuracy. Ridge Regression (RR) shows moderate correlation (r = 0.7002) and normalized coefficient of determination (${R}_{o}^{\mathrm{^{\prime}}2}$=0.9195). However, the model's Nash–Sutcliffe Efficiency (Rm = 0.3859) and Willmott's index (m = − 0.7006) indicate lower accuracy compared to some other models. Support Vector Machine (SVM) exhibits moderate correlation (r = 0.8179) and normalized coefficient of determination (${R}_{o}^{\mathrm{^{\prime}}2}$=0.9464). The Nash–Sutcliffe Efficiency (Rm = 0.5850) and Willmott's index (m = − 0.3308) suggest reasonable accuracy. K-Nearest Neighbors (KNN) shows high correlation (r = 0.8719) and normalized coefficient of determination (${R}_{o}^{\mathrm{^{\prime}}2}$=0.9606), indicating good predictive power. The Nash–Sutcliffe Efficiency (Rm = 0.6939) and Willmott's index (m = − 0.2100) support its accuracy. AdaBoost (AB) exhibits high correlation (r = 0.9529) and normalized coefficient of determination (${R}_{o}^{\mathrm{^{\prime}}2}$=0.8517). The Nash–Sutcliffe Efficiency (Rm = 0.7150) and Willmott's index (m = 0.3964) indicate reasonably good accuracy. Random Forest (RF) shows strong correlation (r = 0.9233) and normalized coefficient of determination (${R}_{o}^{\mathrm{^{\prime}}2}$=0.9412). The Nash–Sutcliffe Efficiency (R_m_ = 0.8432) and Willmott's index (m = -0.0240) suggest high accuracy. Voting Regressor (VR) exhibits high correlation (r = 0.9696) and normalized coefficient of determination (${R}_{o}^{\mathrm{^{\prime}}2}$=0.9628). The Nash–Sutcliffe Efficiency (Rm = 0.9308) and Willmott's index (m = 0.0205) suggest accurate predictions. Gradient Boosting (GB) shows very high correlation (R = 0.9727) and normalized coefficient of determination (${R}_{o}^{\mathrm{^{\prime}}2}$=0.9632). The Nash–Sutcliffe Efficiency (Rm = 0.9340) and Willmott's index (m = 0.0260) indicate excellent predictive accuracy. XGBoost (XGB) exhibits very high correlation (r = 0.9791) and normalized coefficient of determination (${R}_{o}^{\mathrm{^{\prime}}2}$=0.9583). The Nash–Sutcliffe Efficiency (Rm = 0.9342) and Willmott's index (m = 0.0516) suggest outstanding predictive power. In summary, XGBoost stands out as the most accurate model, closely followed by Gradient Boosting and CatBoost. These models demonstrate strong correlations and high coefficients of determination, indicating their ability to accurately predict the target variable. However, each model has its strengths and weaknesses, and the choice of the most suitable model should consider the specific requirements and nuances of the research problem at hand.

**Table 7:** Parameters of external validation of AI models.

Model	r	k	${k}{\prime}$	${R}_{o}^{2}$	${R}_{o}^{{\prime}2}$	R_m_	m	n
CB	0.9753	0.9810	1.01022	0.9597	0.9631	0.9364	0.0317	0.0248
LR	0.7232	0.8526	1.07444	0.7411	0.8233	0.5137	-0.0500	− 0.2959
ANN	0.8749	0.9403	1.01945	0.9139	0.9245	0.7356	− 0.0912	− 0.1165
DT	0.9054	0.9692	1.0000	0.9692	0.9701	0.7674	− 0.1460	− 0.1481
RR	0.7002	0.9131	1.0000	0.9131	0.9195	0.3859	− 0.7006	− 0.7244
SVM	0.8179	0.9436	1.0000	0.9436	0.9464	0.5850	− 0.3308	− 0.3388
LR	0.6844	0.8815	1.0315	0.8452	0.8723	0.3931	− 0.5249	− 0.6241
KNN	0.8719	0.9591	1.0000	0.9591	0.9606	0.6939	− 0.2100	− 0.2139
AB	0.9529	0.9356	1.0517	0.7403	0.8517	0.7150	0.3964	0.2010
RF	0.9233	0.9580	1.0157	0.9344	0.9412	0.8432	− 0.0240	− 0.0391
VR	0.9696	0.9791	1.0102	0.9596	0.9628	0.9308	0.0205	0.0139
GB	0.9727	0.9802	1.0102	0.9599	0.9632	0.9340	0.0260	0.01938
XGB	0.9791	0.9809	1.0114	0.9535	0.9583	0.9342	0.0516	0.0420

The extensive evaluation of thirteen machine learning models focused on predicting the compressive strength of preplaced aggregate concrete (PAC) has unveiled crucial practical applications across diverse industries and research spheres. Firstly, models like XG-Boost, Gradient Boosting, and Cat Boost exhibit exceptional accuracy, empowering engineers to optimize concrete mixtures by adjusting proportions of key components while minimizing material waste. Secondly, these models enhance quality control in construction by enabling real-time strength predictions, ensuring compliance with standards and bolstering safety measures. Additionally, their external validation supports cost-efficient material management, aiding in procurement optimization and minimizing unnecessary expenses. Thirdly, insights derived from these models facilitate tailored structural designs to meet specific project requirements while optimizing material usage. Finally, these findings serve as a guiding light for future research, encouraging innovation in concrete technology. Overall, the practical utility of machine learning models extends far beyond academia, promising precise predictions and invaluable insights that can revolutionize concrete production, construction practices, and structural design within the construction industry.

### SHAP analysis

SHAP Analysis, rooted in the work of^103^, offers a comprehensive methodology for interpreting machine learning models through the introduction of Shapely Additive explanations. In essence, Shapely values serve as a pivotal technique for elucidating the relative impact and contribution of each input variable toward the ultimate output variable. This concept aligns with parametric analysis, where all variables are held constant except for one, thereby allowing an exploration of the effect of the altered variable on the target attribute. This section is dedicated to the assessment of the relative importance of each variable, thereby delineating the influence of input variables on the compressive strength of preplaced aggregate.

It is imperative to underscore that, in the present context, the XGBoost model outperforms other models in terms of accuracy. Consequently, XGBoost has been selected as the model of choice for conducting the SHAP Analysis.

**Figure 19:**
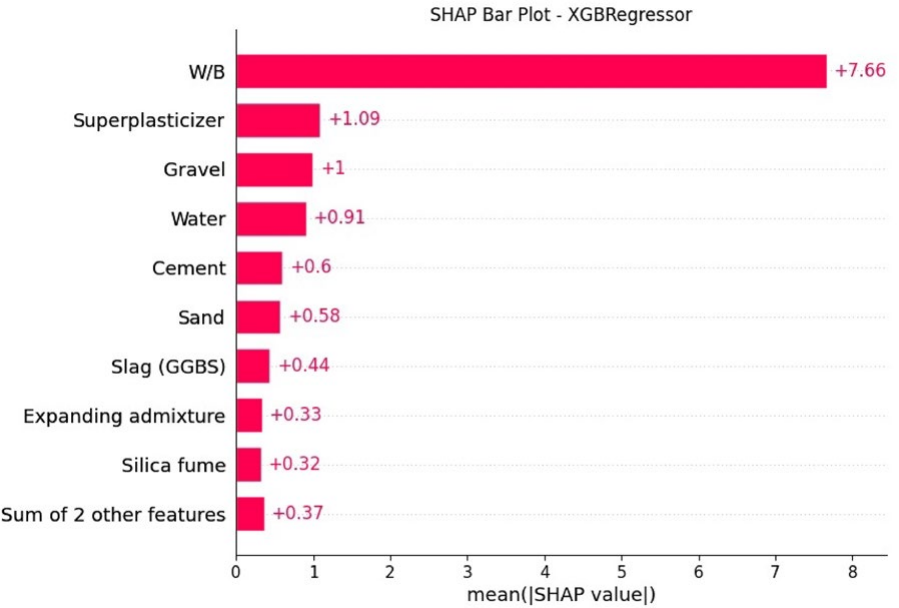
SHAP Analysis.

The mean SHAP values, derived from the Shapely Additive explanations, furnish invaluable insights into the nuanced impact of various input features on the compressive strength of preplaced aggregate concrete. These values play a pivotal role in ascertaining the relative significance of each feature, offering a comprehensive understanding of their contributions to the model's output. The analysis focuses on the mean SHAP values for specific features crucial to the concrete's compressive strength as indicated in Fig. 19. The Fly Ash and Sand to Binder Ratio (S/B) at 0.37 demonstrate a noteworthy positive impact on compressive strength, implying that an elevated ratio positively influences the concrete's strength. Silica Fume, with a SHAP value of 0.32, exhibits a similarly positive influence on compressive strength, indicating the advantageous role of higher silica fume proportions. Ground Granulated Blast-furnace Slag, marked by a SHAP value of 0.44, accentuates its favorable impact on compressive strength, underlining the significance of increased slag content. Sand, with a SHAP value of 0.58, emerges as a pivotal contributor to compressive strength, reflecting the positive influence of higher sand content. Water, with a notably high SHAP value of 0.91, stands out as a significant factor, emphasizing its critical role and potential sensitivity to the concrete's compressive strength. Gravel, denoted by a SHAP value of 1, establishes an essential and direct correlation with compressive strength, indicating substantial impacts with variations in gravel content. The water to binder ratio (W/B) exhibits a considerable SHAP value of 7.66, underscoring its pivotal role and emphasizing the sensitivity of compressive strength to variations in this ratio. Superplasticizer, with a SHAP value of 1.09, demonstrates a positive influence on compressive strength, emphasizing the constructive role of higher superplasticizer content. Cement, marked by a SHAP value of 0.6, emerges as a key determinant, highlighting the importance of optimal cement content for achieving desired strength. The Expanding Admixture, with a SHAP value of 0.33, indicates a positive contribution to compressive strength, enhancing the overall strength characteristics of the concrete. In summary, the comparison of mean SHAP values provides a nuanced understanding of the relative importance of each variable. Features with higher SHAP values, such as water, gravel, and the water-to-binder ratio, exert substantial influence, underscoring the need for meticulous consideration and precise control of these variables in optimizing compressive strength. Conversely, variables with lower SHAP values, such as silica fume and fly ash, remain influential but may not carry as much weight in comparison.

### Grapgical user interface

**Figure 20:**
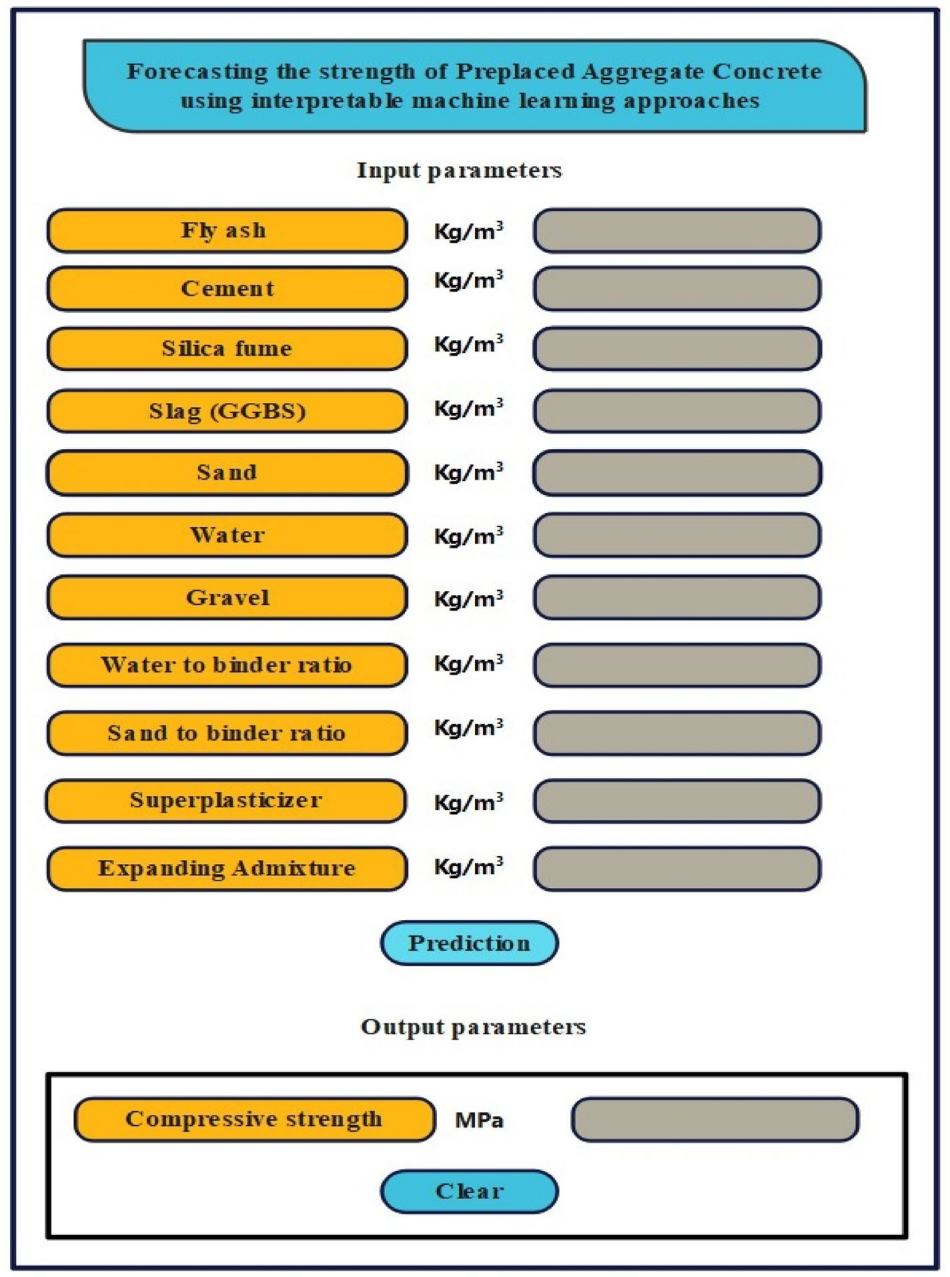
Graphical User Interface model.

A computer application featuring a user-friendly interface was developed to predict compressive strength, as illustrated in Fig. 20. Users can input parameter values and generate output through a simple interface, making it advantageous for both academic research and industrial applications. The error handling functionality in the proposed graphical user interface (GUI) ensures users input values within an acceptable range, facilitating the retrieval of mechanical strength characteristics. The authors propose that this approach has the potential to provide a convenient and efficient method for predicting strength characteristics. Consequently, the use of a GUI can be a significant aid for practitioners and engineers with limited expertise in complex programming languages, enabling them to implement machine-learning models effectively.

## Conclusion

- In this study, 13 machine learning (ML) models were examined using an extensive dataset comprising 2871 experimental records to forecast the compressive strength of preplaced aggregate concrete (PAC). The critical factors influencing compressive strength included fly ash, silica fume, Ground Granulated Blast-furnace Slag, sand, water, gravel, Water to binder ratio (W/B), Sand to binder ratio (S/B), superplasticizer, and expanding admixture. The internal complex responses depending on the presence of different ingredients on the overall compressive strength of PAC are explored. ML models: linear regression (LR), lasso regression (Lasso), ridge regression (RR), support vector machine (SVM), artificial neural network (ANN), k-nearest neighbours (KNN), decision tree (DT), AdaBoost (AB), random forest (RF), Voting Regressor (VR), Cat Boost (CB), Gradient Boost (GB) and XG Boost (XGB) were trained using the input parameters.
- The comprehensive analysis of various machine learning models in predicting the compressive strength of preplaced aggregate concrete reveals valuable insights for practical applications. Among the models evaluated, XG Boost emerges as the most accurate, demonstrating exceptional predictive power with a correlation coefficient of 0.9791 and a normalized coefficient of determination (${R}_{o}^{\mathrm{^{\prime}}2}$) of 0.9583. Following closely are Gradient Boosting and CatBoost, both displaying strong correlations and high coefficients of determination.
- These models, alongside AdaBoost, Voting Regressor, and Random Forest, showcase precise predictions with low mean absolute error (MAE) and root mean square error (RMSE) values, indicating their efficacy in capturing intricate data patterns. In contrast, linear regression, ridge regression, and K-nearest neighbors exhibit comparatively higher prediction errors, emphasizing the significance of choosing appropriate models for accurate predictions.
- The external validation results further corroborate the effectiveness of models like Cat Boost, ANN, and Decision Tree, showcasing their ability to explain the variance in concrete compressive strength accurately.
- The sensitivity analysis chart reveals key contributors to the model's sensitivity. Gravel commands the highest sensitivity at 44.7%, underscoring its pivotal role. Sand also stands out with 19.5%, emphasizing its profound influence on the model's sensitivity.
- SHAP Analysis, based on Shapely Additive explanations, interprets machine learning models by revealing the relative impact of input variables. XGBoost, chosen for its superior accuracy, undergoes SHAP Analysis to understand its contribution. Mean SHAP values highlight key factors like Water to Binder Ratio, Gravel, and Water, underscoring their substantial influence on optimizing compressive strength in preplaced aggregate concrete.
- This comprehensive evaluation provides researchers and practitioners with valuable guidance, enabling them to make informed decisions tailored to specific research contexts, thereby enhancing the reliability and applicability of predictive models in the realm of preplaced aggregate concrete strength prediction.

## Data Availability

The datasets used and/or analyzed during the current study available from the corresponding author on reasonable request.

## Abbreviations

ITZ: Interfacial transition zones
PAC: Preplaced-aggregate concrete
HPC: High-performance concrete
CS: Compressive strength
W/B: Water to binder ratio
FA: Fly ash
SF: Silica fume
GGBFS: Ground granulated blast-furnace slag
S/B: Sand to binder ratio
LR: Linear regression
RR: Ridge regression
DT: Decision tree
RF: Random forest
SVR: Support vector regression
ANN: Artificial neural network
KNN: K-nearest neighbors algorithm
AB: Ada-boost
CBR: Cat-boost regressor
VR: Voting regression
GB: Gradient boost
XGB: Extreme gradient boosting machine
RHAC: Rice husk ash concrete
AR: AutoRegressive models
GEP: Gene expression programming
MLR: Multiple linear regression
RSM: Response surface methodology
PSO: Particle swarm optimization
